# Targeting HIF2α-ARNT hetero-dimerisation as a novel therapeutic strategy for pulmonary arterial hypertension

**DOI:** 10.1183/13993003.02061-2019

**Published:** 2021-03-04

**Authors:** David Macias, Stephen Moore, Alexi Crosby, Mark Southwood, Xinlin Du, Huiling Tan, Shanhai Xie, Arlette Vassallo, Alexander J.T. Wood, Eli M. Wallace, Andrew S. Cowburn

**Affiliations:** 1 CRUK Cambridge Centre Early Detection Programme, Dept of Oncology, Hutchison/MRC Research Centre, University of Cambridge, Cambridge, UK; 2 Dept of Medicine, University of Cambridge, Cambridge, UK; 3 Dept of Pathology, Papworth Hospital National Health Service Foundation Trust, Cambridge, UK; 4 Peloton Therapeutics Inc. (a subsidiary of Merck & Co., Inc., Kenilworth, NJ, USA), Dallas, TX, USA; 5 National Heart and Lung Institute, Imperial College London, London, UK; 6 Both authors contributed equally

## Abstract

Pulmonary arterial hypertension (PAH) is a destructive disease of the pulmonary vasculature often leading to right heart failure and death. Current therapeutic intervention strategies only slow disease progression. The role of aberrant hypoxia-inducible factor (HIF)2α stability and function in the initiation and development of pulmonary hypertension (PH) has been an area of intense interest for nearly two decades.

Here we determine the effect of a novel HIF2α inhibitor (PT2567) on PH disease initiation and progression, using two pre-clinical models of PH. Haemodynamic measurements were performed, followed by collection of heart, lung and blood for pathological, gene expression and biochemical analysis. Blood outgrowth endothelial cells from idiopathic PAH patients were used to determine the impact of HIF2α-inhibition on endothelial function.

Global inhibition of HIF2a reduced pulmonary vascular haemodynamics and pulmonary vascular remodelling in both su5416/hypoxia prevention and intervention models. PT2567 intervention reduced the expression of PH-associated target genes in both lung and cardiac tissues and restored plasma nitrite concentration. Treatment of monocrotaline-exposed rodents with PT2567 reduced the impact on cardiovascular haemodynamics and promoted a survival advantage. *In vitro*, loss of HIF2α signalling in human pulmonary arterial endothelial cells suppresses target genes associated with inflammation, and PT2567 reduced the hyperproliferative phenotype and overactive arginase activity in blood outgrowth endothelial cells from idiopathic PAH patients. These data suggest that targeting HIF2α hetero-dimerisation with an orally bioavailable compound could offer a new therapeutic approach for PAH. Future studies are required to determine the role of HIF in the heterogeneous PAH population.

## Abstract 2

**PAH is a debilitating disease with no cure. There is an unmet need for new transformative therapies. Targeting HIF2α function through inhibiting ARNT hetero-dimerisation reduces many clinical symptoms associated with established PH disease in animals.** https://bit.ly/3jHK8PS

## Introduction

Oxygen exchange in the lungs requires a fine matching between ventilation and perfusion. Although the lungs are exposed to the highest partial oxygen tension in the body, there are physiological and pathological conditions that result in local low oxygen availability (hypoxia). This local hypoxia is counteracted by the exclusive, unique oxygen-sensing capability of the pulmonary vasculature characterised by a profound vasoconstrictive response to diminishing oxygen tension [1]. Regional lung vasoconstriction stimulates a dynamic shift in perfusion to aid maximal capture of oxygen; however, prolonged exposure to hypoxia initiates a potent stimulus that leads to pulmonary vascular remodelling that is a hallmark of idiopathic pulmonary fibrosis [2], chronic obstructive pulmonary disease [3] and pulmonary hypertension [4]. In pulmonary arterial hypertension (PAH), changes in endothelial cell and smooth muscle cell proliferation, apoptosis and metabolism result in vascular remodelling and obstruction. The resulting phenotype progressively reduces pulmonary vascular plasticity, identity and compliance, thereby increasing pulmonary vascular resistance (PVR) and back-pressure on the right ventricle (RV), leading to RV hypertrophy and ultimately right-heart failure and death [2]. Currently, there is no cure for PAH, with all existing therapies only slowing disease progression with limited impact on mortality. Therefore, there is an unmet clinical need to develop novel transformative therapies.

This programmed response to chronic hypoxia is in part regulated by hypoxia-inducible factors (HIFs) that belong to a group of basic helix-loop-helix-PER-ARNT-SIM (PAS) proteins that function as transcription factors responding to oxygen and other stress [5]. The HIFs function as heterodimers composed of a constitutively expressed β-HIF-1b/ARNT subunit and an oxygen-regulated α-subunit that includes HIF1α and HIF2α. Each of the subunits contains two PAS domains (PAS-A and PAS-B) that contribute to the stability of the α- and β-heterodimer complex. HIFα activity is controlled post-translationally by the concerted action of oxygen, prolyl-hydroxylases (PHDs) and the ubiquitin proteasome system. In normoxia, PHDs hydroxylate HIFα, enabling the ubiquitin proteasome system to bind and rapidly degrade the protein. Under hypoxia, inactive PHDs allow HIFα proteins to accumulate and dimerise with ARNT to form an active transcription factor complex. In addition, HIFα stability and function can be influenced by aberrantly active cytokines, growth factors, metabolites and reduced PHD expression, independent of oxygen, resulting in a pseudo-hypoxia phenotype [6–9]. Both of these HIFα regulatory pathways contribute to the initiation and progression of idiopathic PAH [10, 11].

Both HIF1α and HIF2α isoforms have been extensively studied in pulmonary hypertension (PH). The first direct evidence came from mice hemizygous for either *HIF1*α [12] or *HIF2*α [13]. Pulmonary disease progression following chronic hypoxia exposure was substantially delayed in these models. Subsequent studies identified tissue-specific HIFα expression in the pulmonary vasculature where HIF2α was found to be highly expressed in the endothelium [14, 15]. We reported that genetic ablation of pulmonary endothelial HIF2α prevented the initiation and development of pulmonary vascular remodelling associated with chronic hypoxia-induced PH [16]. Several groups have now reported that endothelial loss of *PHD2* in mice leads to the aberrant stability of HIF2α with the development of occlusive vascular lesions and severe PH. The concomitant genetic ablation of endothelial *PHD2* and *HIF2*α in this model of PH also inhibited the phenotype, offering near-complete protection from PH [8, 9]. These murine gene manipulation studies established HIF2α as the predominant HIFα isoform driving PH. Moreover, patients or mice with HIF2α gain-of-function mutation have elevated pulmonary haemodynamics associated with PH, further demonstrating that aberrant stability of HIF2α can initiate this disease [17, 18]. Although substantial progress has been made unravelling the role of HIF2α in PH, it has taken almost two decades to identify and develop suitable small-molecule inhibitors as potential candidates for PH therapy.

As a transcription factor that activates gene expression through protein–protein interactions, HIF2α was generally regarded as intractable for small-molecule inhibition. However, biophysical studies by Scheuermann *et al.* [19] led to the discovery that the inner core of the PAS-B domain of HIF2α possesses a hydrophobic cavity that can bind small molecules that allosterically disrupt its dimerisation to ARNT and thereby block transcriptional activity. Independently, Zimmer *et al.* [20] used a cellular screening approach to identify HIF2α inhibitors that resulted in the discovery of compound 76 (C76). C76 inhibits HIF2α translation by binding to the iron regulatory protein. While the latter approach led to a molecule that has been used in a handful of pre-clinical models, it demonstrates only micromolar potency *in vitro*, and has not been shown to be orally bioavailable. In contrast, the former approach led to a series of highly selective, orally bioavailable HIF2α inhibitors. These direct HIF2α inhibitors are efficacious in rodent cancer models, and, importantly, demonstrate antitumour activity in metastatic clear-cell renal cell carcinoma (ccRCC) patients along with a favourable safety and tolerability profile [21–25]. As such, given that HIF2α is a potent stimulus of pulmonary vascular remodelling in PH, we evaluated a direct HIF2α inhibitor, PT2567 [25] in pre-clinical PH models. We performed haemodynamic profiling and biochemical analysis of the rodent su5416/hypoxia model using both prevention and intervention strategies with vehicle, PT2567 or sildenafil as a clinically relevant comparative compound. Herein we demonstrate that HIF2α inhibition reduces haemodynamic parameters associated with severe PH development by reversing pulmonary vascular remodelling, decreasing circulating pro-inflammatory factors and by restoring plasma nitrite levels, suggesting that inhibition of this critical pathway could provide a promising new strategy in the treatment of PH.

## Methods

### Animals

This research has been regulated under the United Kingdom Animals (Scientific Procedures) Act 1986 amendment regulations 2012 following ethical review by the University of Cambridge animal welfare and ethical review body (AWERB) Home Office project licence 70/8850. All animals were housed on a 12 h/12 h light/dark cycle with *ad lib* standard chow and water. A power calculation determined the number of rats required per group to detect a 25% effect change at 80% power, 5% significance level. The group size for the su5416/hypoxia prevention studies was calculated to be n=10, and the su5416/hypoxia and monocrotaline (MCT) intervention studies were calculated to be n=15 and n=14, respectively

### Sugen5416/hypoxia rat model of PH

Male Sprague Dawley rats (∼150–200 g; Charles River, Wilmington, MA, USA) were given a single subcutaneous injection of su5416 (20 mg·kg^−1^; Tocris, Bristol, UK) in vehicle (0.5% carboxyl methylcellulose sodium, 0.4% polysorbate 80, 1% benzyl alcohol 5% dimethyl sulfoxide (DMSO) 5% polyethylene glycol 400; all Sigma, Gillingham, UK), placed immediately into a 10% oxygen (O_2_) chamber and maintained in hypoxia for 3 weeks. Depending on the treatment strategy, rats assigned to the prevention protocol were randomly assigned to four treatment groups and received vehicle, PT2567 (100 mg·kg^−1^ or 300 mg·kg^−1^) by oral gavage once daily or sildenafil (30 mg·kg^−1^) twice daily during the hypoxic exposure. Rats assigned to the intervention protocol were allowed to acclimate to normoxia for 24 h before being randomly assigned to three groups for treatment with vehicle, PT2567 (100 mg·kg^−1^) by oral gavage twice daily and sildenafil (30 mg·kg^−1^) by oral gavage twice daily for 3 weeks. Cardiac echo, right ventricle systolic pressure and right ventricle hypertrophy were measured, as described previously [26–28]. Cardiac echo parameters were measured using a (Vevo 3100 Imaging System; Fujifilm VisualSonics Inc., Toronto, Canada). A parasternal long-axis view of the left ventricle (LV) was obtained in order to visualise the LV, aorta and mitral valve leaflets. The pulmonary artery was visualised and colour Doppler confirmed correct placement of a pulse-wave Doppler within the maximum flow velocity. Peak velocity of the pulmonary artery was measured to determine pulmonary outflow along with the right-ventricle outflow tract velocity time interval (RVOT-VTI). Pulmonary acceleration time (PA-AT, defined as the time from the onset of flow to peak velocity by pulsed-wave Doppler recording) and right-ventricular ejection time (PA-ET, the time from the onset to the termination of pulmonary flow) were measured. An apical four-chamber view was obtained to quantify the blood flow spectrum and characterise peak tricuspid regurgitation velocity (TRV) (if present). An estimate of PVR (Woods units (WU)) was calculated using the formula PVR=10×TRV/RVOT-VTI. All echocardiograms were performed by the same individual and analysis was performed offline and the individual was blinded to the treatment group. Afterwards, heart, lung and blood were taken for histological, gene expression and biochemical analysis.

### MCT rat model of PH

Male (Sprague Dawley) rats (100–125 g; Charles River) were randomly allocated to four groups and injected *s.c.* with either vehicle control (0.9% saline) at 2 mL·kg^−1^ or 40 mg·kg^−1^ (2 mL·kg^−1^) MCT (PHL89251; Sigma-Aldrich) as described previously [26]. 14 days post-MCT or vehicle injection animals underwent a baseline cardiac echo (Vevo 3100 Imaging System) in order to ascertain disease severity. Echocardiograms were performed by the same individual and blinded to the treatment groups. All analysis was performed offline. The animal treatment groups were as follows. Group 1: vehicle nondisease control (carboxymethyl cellulose in 0.9% saline); group 2: vehicle disease control (carboxyl methylcellulose in 0.9% saline); group 3: HIF2α-inhibitor intervention (PT2567 100 mg·kg^−1^) oral gavage twice daily; group 4: sildenafil intervention (30 mg·kg^−1^) by oral gavage twice daily for 2 weeks. Following the dosing period, all animals underwent cardiac echocardiography, as described earlier.

### Pulmonary vascular morphometry

Lung were inflated and fixed *via* the trachea with 4% paraformaldehyde at a constant fluid pressure of 15–20 cm for 5 min. Lungs from the prevention protocol were stained for elastin/eosin, and those from the intervention protocol were stained with haematoxylin and eosin, elastic Van Gieson (EVG) stain to assess histology (MSD/BDH, Lutterworth, UK). Histological findings were assessed by a blinded independent pathologist who scored the lung sections for perivascular/vascular inflammation, perivascular fibrosis and smooth-muscle hypertrophy of small arterioles. The findings were recorded as 0 (normal), 1 (minimal), 2 (mild), 3 (moderate), 4 (marked). Scoring of smooth muscle hypertrophy in pulmonary arterioles was based on thickness of the muscle wall and the extent of the finding (apparent number of arterioles affected). In addition, serial lung sections from the intervention protocol were immunostained with anti-smooth muscle α-actin (α-SM actin; DakoCytomation, Ely, UK), von Willebrand factor and anti- myeloperoxidase (MPO) (DakoCytomation), and anti-Ki67 (Abcam, Cambridge, UK) to assess the degree of muscularisation of small pulmonary arteries (≤50 μm), cellular proliferation and MPO-positive cells. Antibody staining was visualised using 3–3′ diaminobenzidine hydrochloride substrate (DakoCytomation) and counterstained with Carrazzi haematoxylin (Bios, Shelmersdale, UK). Vessel medial thickness was measured using Image J software (MediaCybernetics, Bethesda, MD, USA). Tissue samples were independently coded and quantified by a blinded pathologist.

### Haematological analysis

Anticoagulated blood was analysed using Vet abc haematology analyser (Horiba, Kyoto, Japan) according to the manufacturer's instructions. Cytokine and growth factor profiling was undertaken in plasma isolated from anticoagulated whole blood which had undergone centrifugation at 1500×*g* for 5 min and frozen to −80°C.

### Nitrite analysis

Blood samples were centrifuged to separate plasma and were passed through a column with a 10-kDa cut-off filter. All samples were analysed for nitrite content using a NOA 280i (Sievers, GE Healthcare, Chicago, IL, USA) according to the manufacturer's instructions.

### RNA analysis

Total RNA was isolated from tissues using TRI-reagent (Sigma) followed by RNA clean-up and DNase digest using RNeasy column kits (Qiagen, Venlo, the Netherlands). First-strand synthesis was performed with 1 μg of total RNA using Superscript IV (Invitrogen, Waltham, MA, USA) according to the manufacturer's instructions. Relative gene expression was determined using quantitative (q)PCR (One-Step Plus Real-Time PCR System; Life Technologies, Waltham, MA, USA) and was amplified in SYBR-Green master mix (Roche, Basel, Switzerland) and relevant primers from Qiagen. Relative gene-expression levels were corrected to housekeeping genes β-actin and B_2_M.

### Carotid body histology

The carotid bifurcation was dissected, fixed for 2 h with 4% paraformaldehyde (Santa Cruz Biotechnology, Dallas, TX, USA) and cryopreserved (30% sucrose in PBS) for cryosectioning (10 μm thick; Bright Instruments, Luton, UK). Tyrosine hydroxylase (TH), was detected using rabbit anti-TH (1:5000; Novus Biologicals, Abingdon, UK; NB300-109) primary antibody and Alexa-Fluor 568-conjugated anti-rabbit IgG (1:500; ThermoFisher, Waltham, MA, USA).

Carotid body area was measured on micrographs (Leica DM-RB, Wetzlar, Gernany) taken from sections spaced 60 µm apart across the entire carotid body using Fiji Software [29]. Carotid body volume was estimated according to Cavalieri's principle, as reported previously [30]. Tissue samples were coded and quantified by a blinded investigator.

### PT2567 Sprague Dawley rat pharmacokinetics

PT2567 was suspended in methylcellulose Tween-80 (0.5% methylcellulose, 0.5% Tween-80 in water) and rats (n=3 per dose level) were dosed by oral gavage. Blood samples were taken at 0.25, 0.5, 1, 2, 4, 8, 12 and 24 h post-dose. Plasma PT2567 concentrations were conducted by non-compartmental method using Pharsight WinNonlin (Certara, Princeton, NJ, USA). A non-compartmental model was used to analyse the data.

### Blood outgrowth endothelial cell isolation culture

Blood outgrowth endothelial cells (BOECs) were isolated from the blood of participants previously diagnosed with PAH or from normal, healthy volunteers at the Addenbrooke's teaching hospital Cambridge University Hospitals NHS Foundation Trust (Cambridge, UK), following a protocol approved by the Cambridge research ethics committee (REF:11/EE/0297). Table 1 contains characteristics of PAH patients and information about the healthy volunteers. Mononuclear cells were isolated from 60 mL of venous blood by Ficoll density gradient centrifugation and plated onto type 1 rat tail collagen-coated (BD Biosciences, Franklin Lakes, NJ, USA) flasks in endothelial selective medium (EGM2; Lonza Biologics, Basel, Switzerland) supplemented with 10% embryonic stem cell-qualified fetal calf serum and additional growth factors (EGM2 bullet kit; Lonza Biologics). BOECs appeared after 2–3 weeks and were subsequently passaged when confluent.

**TABLE 1:** Pulmonary arterial hypertension (PAH) patient characteristics for the isolation of blood outgrowth endothelial cells

	**Age** **years**	**Sex**	**Ethnicity**	**mPAP mmHg**	**Cardiac index L·min**^−1^**·m**^−2^	**PVR Woods units**	**6-min walk distance m**	**Treatment**	**PAH class**
**PAH**	45	M	Caucasian	46	1.75	9.7	381	ERA, PDEi	Heritable
**PAH**	79	F	Caucasian	52	2.25	10.96	124	PDEi	Idiopathic
**PAH**	32	M	Caucasian	60	2.69	10.8	400	PGI, PDEi	Heritable
**Control**	30	M	Caucasian						
**Control**	37	F	Caucasian						
**Control**	41	M	Caucasian						

mPAP: mean pulmonary artery pressure; PVR: pulmonary vascular resistance; M: male; ERA: endothelin receptor antagonist; PDEi: phosphodiesterase inhibitor; F; female; PGI: prostacyclin.

BOECs were cultured for a maximum of five passages. For BOEC hypoxic exposure experiments, cells were transferred to a Baker Ruskinn hypoxic chamber (Bridgend, UK) and pre-hypoxic media was transferred into each well with or without inhibitors. Cells were cultured under 1% O_2_ for the experimental times indicated in the results

### Cell culture

786-O and Hep3B cell lines were purchased from ATCC (American Type Culture Collection, Manassas, VA, USA). Cells were cultured in DMEM with 10% fetal bovine serum. Human pulmonary artery endothelial cells (hPAECs) were purchased from PromoCell (Heidelberg, Germany) and cultured in endothelial cell growth medium. For PT2567 treatment cells were plated into six-well plates. PT2567 dissolved in DMSO was added as the cultures reached confluence with a final concentration of DMSO at >0.1%. For hypoxia-treated cells, cell culture media was exposed to 1% O_2_, 5% carbon dioxide (Ruskin) for 12 h before transferring onto cells. Following the addition of PT2567, the cells were maintained under hypoxia for the duration of the treatment.

### Arginase activity assay

BOECs were prepared as described earlier. Urea production was normalised with protein concentration [31].

### Knockdown experiments

Control hPAECs and human BOECs derived from both control volunteers were transduced using lentiviral particles containing three different short-hairpin (sh)RNAs targeting human HIF-1α and HIF-2α mRNA, respectively. shRNA sequences were selected from The RNAi Consortium (TRC) according to the following criteria: shRNAs seeming to have fewer potential off-target binding sites and that were validated by MISSION shRNA Library (Sigma-Aldrich). The individual clone IDs selected were TRCN0000003808 (HIF1α), TRCN0000003806 (HIF2α) and TRCN0000342501. Oligos for each individual shRNA were annealed and cloned into pLKO.1 plasmid following TRC recommendations. Positive colonies were checked by sequencing. To produce lentiviral vectors, Lenti-X 293T cells (Clontech, Saint-Germain-en-Laye, France) were cotransfected with single pLKO.1, pCMV-dR8.91 and pMD2.G plasmids using Lipofectamine 2000 (Thermo-Fisher Scientific) according to the manufacturer's protocol. Lentiviral particles were collected 48 h after transfection and used to transduce hPAECs and BOECs overnight. Next, fresh media was added and the cells were incubated in normoxia for 2 days then in 1% O_2_ atmosphere.

### Proliferation assay

BOECs were plated in 24-well plates at 20 000 cells per well. Cells received complete EGM-2MV medium with/without PT2567. Cells were counted on days, 0, 2, 4 and 6 with trypan blue exclusion.

### BOEC endothelial network formation assay

BOECs from PAH patients and healthy volunteers were grown to 90% confluence. BOECs were transferred into control media or media containing 1 μM PT2567 before seeding at ∼75 000 cells in Matrigel matrix-coated 12-well plates. Images were captured 20 h after treatment using phase-contrast microscopy (Leica MZ16). Tube network length and network loop were determined using Image J software (MediaCybernetics, Bethesda, MD, USA).

### Co-immunoprecipitation of HIF2α and ARNT

Cells in six-well plate were lysed in 1 mL of cell lysis buffer (Tris-HCl 20 mmol·L^−1^, pH 7.5, Triton X-100 1%, NaCl 150 mmol·L^−1^, glycerol 5%, EDTA 1 mmol·L^−1^, dithiothreitol 1 mmol·L^−1^ and one tablet per 10 mL of Roche Protease Inhibitor Tablet Complete). A total of 1 μg of mouse mAb against human ARNT (sc-55526; Santa Cruz Biotechnology) or 1 μg of antibody against HIF2α (Ab199; Abcam) and 50 μL of Protein AG Beads (50% slurry in lysis buffer; Santa Cruz Biotechnology) were added to cleared cell lysate. The tubes were rotated at 4°C for 16 h. After washing in cold lysis buffer, the bead-bound proteins were separated by SDS gel electrophoresis and subjected to Western blotting with specific antibodies.

### Isothermal titration calorimetry

Human HIF2α-B was expressed and purified as described previously [19]. Rat HIF2α differs from human HIF2α-B by only three amino acids. So, the expression vector for human HIF2α-B was mutated at these residues (T262L, I 265V and I326V) to make rat HIF2α-B. The binding affinity between PT2567 and PAS-B domains was determined using isothermal titration calorimetry on an iTC200 system (GE Healthcare). PAS-B at 0.5 mmol·L^−1^ was titrated into 26 μmol·L^−1^ or 25 μmol·L^−1^ of PT2567 for human or rat, respectively, in the cell in buffer consisting of 20 mmol·L^−1^ Tris-HCl, pH 8.0, 150 mmol·L^−1^ KCl, and 1% DMSO.

### Statistical analysis

All data represent the mean±sd of n separate experiments, unless otherwise stated. The difference between groups was assessed using one-way ANOVA with Tukey's multiple comparison test. All data were tested for normal distribution. If not normally distributed, the nonparametric Kruskal–Wallis test was used with Dunn's multiple comparison test, unless otherwise stated. Statistical analysis for the MCT mortality data was assessed by the log-rank (Mantel–Cox) test. Analysis was undertaken using GraphPad Prism (version 7.0 c for Mac OS; San Diego, CA, USA). A p-value of <0.05 was considered significant.

## Results

### HIF2α/ARNT dimerisation disruption

We began by determining the binding affinity of PT2567 for human and rat HIF2α PAS-B domain by isothermal titration calorimetry. The dissociation constant for this HIF2α PAS-B domain interaction is 30 nM and 29 nM for human and rat, respectively (supplementary figure S1a and b). Next, we assessed the disruption of HIF2α/ARNT interaction/dimer by co-immunoprecipitation assay in 786-O cells. This ccRCC cell line was selected, as it constitutively expresses active HIF2α in normoxia without HIF1α due to truncation of the *HIF1*α gene. Initially, we immunoprecipitated ARNT in lysates of 786-O cells treated with PT2567. Immunoblotting shows that ARNT protein co-precipitation with HIF2α is diminished in response to PT2567 treatment in a concentration dependent manner demonstrating disruption of HIF2α/ARNT dimer formation (supplementary figure S1c).

### Inhibition of HIF2α transcription

We evaluated the effect of PT2567 on HIF2α-dependent transcriptional activation of target genes by qPCR analysis. Treatment of 786-O cells with PT2567 significantly reduces the mRNA expression for *GLUT1*, *EPO*, *CCND1*, *PAI1* and *VEGFA*, in a concentration-dependent manner (supplementary figure S2a–e). We further evaluated the specificity of PT2567 in Hep3B hepatoma cells. Hypoxia leads to the accumulation of both HIF1α and HIF2α protein in this cell line. Treatment of Hep3B cells with PT2567 reduced the hypoxic-induced expression of HIF2α target genes only; *EPO* and *PAI1*, with no effect on HIF1α target gene expression; *PDK1* or *PGK1* (supplementary figure S2f–i). These important data confirmed the target specificity of PT2567, only disrupting HIF2α/ARNT interaction without affecting HIF1α/ARNT transcriptional activity.

### PT2567 validation in hPAECs

We next assessed the activity and specificity of PT2567 in primary hPAECs. We initially confirmed HIF1α and HIF2α target genes in these cells through short-hairpin knockdown (shRNA-HIF1α or HIF2α) of these transcription factors followed by timed exposure to hypoxia (supplementary figure S3a–f). We confirmed the near-complete inhibition of PAI-1 and partial inhibition of VEGF and GLUT1 mRNA expression in HIF2α^−/−^ PAECs with no effect on HIF1α target LDHA mRNA expression. Treatment of hPAECs with PT2567 markedly reduced the hypoxia-induced expression of HIF2α target genes and PH-associated genes: *GLUT1*, *VEGF*, *CXCL12*, *CXCR4*, *ICAM1*, *Sele*, *PAI1* and *APLN*, and restored *ID1* gene expression in a concentration-dependent manner without effecting HIF1α target gene transcripts for *LDHA* and *PDK1* (supplementary figure S3g–q). These results confirm that PT2567 disrupts HIF2α/ARNT interaction without affecting HIF1α/ARNT transcriptional activity in hPAECs.

### PT2567 prevents the initiation and development of PH

**FIGURE 1:**
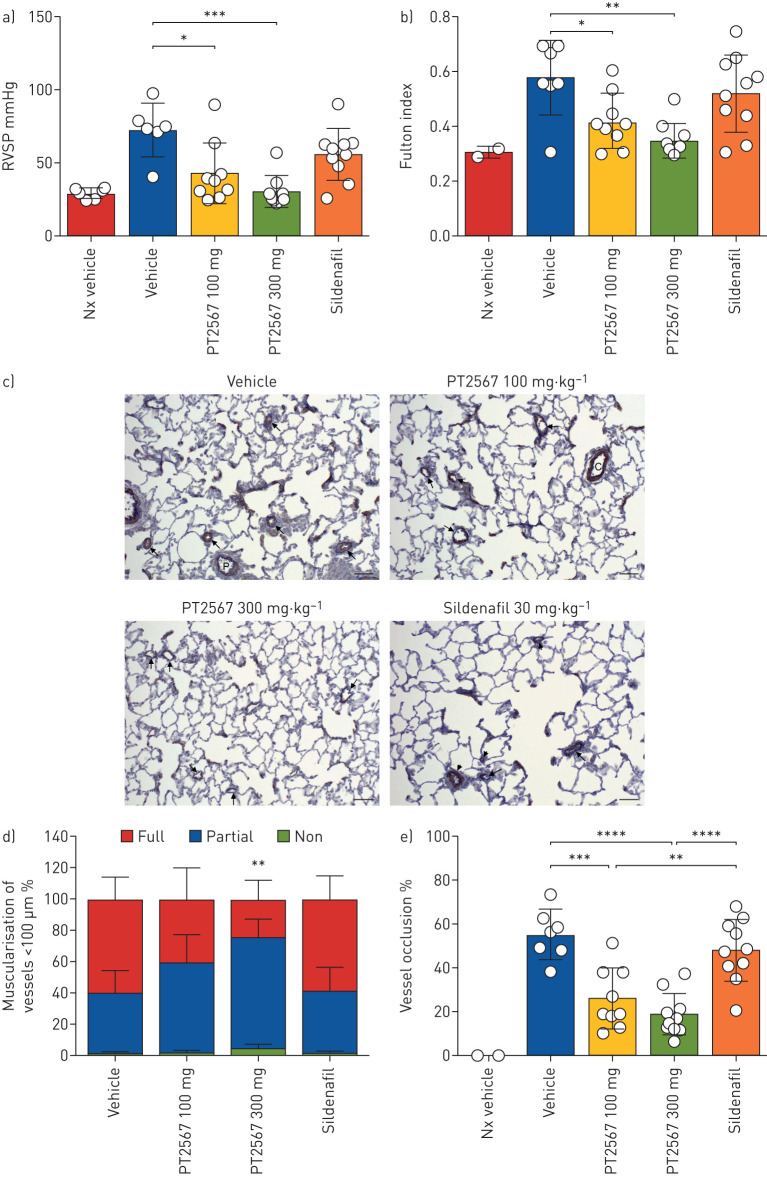
Effect of hypoxia-inducible factor (HIF)2α-inhibitor on the development of pulmonary arterial hypertension (PAH) in a rat SU-5416/Hx prevention model. a) Right ventricular systolic pressure (RVSP); b) right ventricle hypertrophy (RVH) showing Fulton index (right ventricle/left ventricle+septum weight ratio) in Sprague Dawley Su5416/Hx rats gavaged once daily with vehicle (n=7), PT2567 100 mg·kg^−1^ (n=9) or 300 mg·kg^−1^ (n=9), or 30 mg·kg^−1^ sildenafil (n=10); c) representative photomicrographs immune-stained for α-smooth muscle actin; arrows point to (vehicle) completely muscularised vessels; (PT2567 100 mg·kg^−1^ and 300 mg·kg^−1^) partially muscularised vessels; (sildenafil) short arrow: completely muscularised, long arrow: partially muscularised distal vessels; scale bar=50 μm; d) stacked-bar chart showing quantification of muscularisation of peripheral pulmonary vessels in lung sections (no smooth muscle ring, smooth muscle ring and full smooth muscle ring); e) determination of small arterial vessel (>5 μm) occlusion, shown as percentage fully occluded. Data are presented as mean±sd. *: p<0.05, **: p<0.001, ***: p<0.0001, ****: p<0.00001 (one-way ANOVA).

We next determined the rat plasma pharmokinetic profiles for PT2567. Timed plasma analysis identified both 100 mg·kg^−1^ and 300 mg·kg^−1^ oral gavage achieved good exposure (supplementary figure S4a). We initially investigated the effect of PT2567 on PH development with a prevention protocol using the rat su5416/Hx model. Rats were randomly assigned to four treatment groups, received a single *s.c.* dose of su5416 and were then housed in 10% O_2_ for 4 weeks. The rats received vehicle, PT2567 (100 mg·kg^−1^ or 300 mg·kg^−1^) once daily or sildenafil (30 mg·kg^−1^) twice daily by oral gavage (supplementary figure S4b). The development and progression of pulmonary hypertension was assessed through measurement of right ventricular systolic pressure (RVSP). RVSP in both PT2567 treatment groups was significantly lower, in a dose-dependent manner (43.22±20.72 mmHg and 30.78±11.07 mmHg for 100 and 300 mg·kg^−1^ groups, respectively) compared to vehicle (72.83±18.14 mmHg). In contrast, sildenafil treatment only showed a modest blunting of RVSP, not significantly different from vehicle (56.10±17.55 mmHg) (figure 1a). In addition, the Fulton index (RV/LV+septum), as an indicator of right ventricle hypertrophy (RVH), was lower in PT2567-treated animals (0.421±0.100 and 0.350±0.06 for 100 and 300 mg·kg^−1^ groups, respectively) when compared to vehicle (0.578±0.13) (figure 1b). Sildenafil treatment provided only little protection from RVH (0.5220±0.14) (figure 1b). Consistent with the data described earlier, there is a positive correlation between RVSP and RVH (r^2^=0.666, p<0.0001) (supplementary figure S4c). Final body weight across all treatment groups did not deviate from vehicle controls (supplementary figure S4d).

Assessment of pulmonary histopathology is presented in supplementary table S1a. Smooth muscle hypertrophy associated with distal pulmonary vessels (≤50 μm), perivascular/vascular inflammation and perivascular fibrosis scoring was greatest in the vehicle-treated group (5.42); meanwhile, there was a dose-dependent decreased score in animals treated with PT2567 100 mg·kg^−1^ (3.66) and 300 mg·kg^−1^ (2.55). The sildenafil group scored higher (4.00) than PT2567-treated animals. Additionally, we quantified vessel muscularisation and observed a decreased percentage of fully muscularised arterioles accompanied by an increase in nonmuscularised arterioles in animals treated with PT2567 compared with vehicle- or sildenafil-treated groups (figure 1c and d and supplementary figure S4e–g).

Next, we assessed the extent of small arterial occlusion. Vessels (≤50 μm) were determined to be either fully occluded or with an open lumen. We observed a decrease in the percentage of occluded vessels in both PT2567- (100 mg·kg^−1^ and 300 mg·kg^−1^) treated groups compared to vehicle- and sildenafil-treated groups (figure 1e).

### Intervention with PT2567 decreases PVR in established PH

**FIGURE 2:**
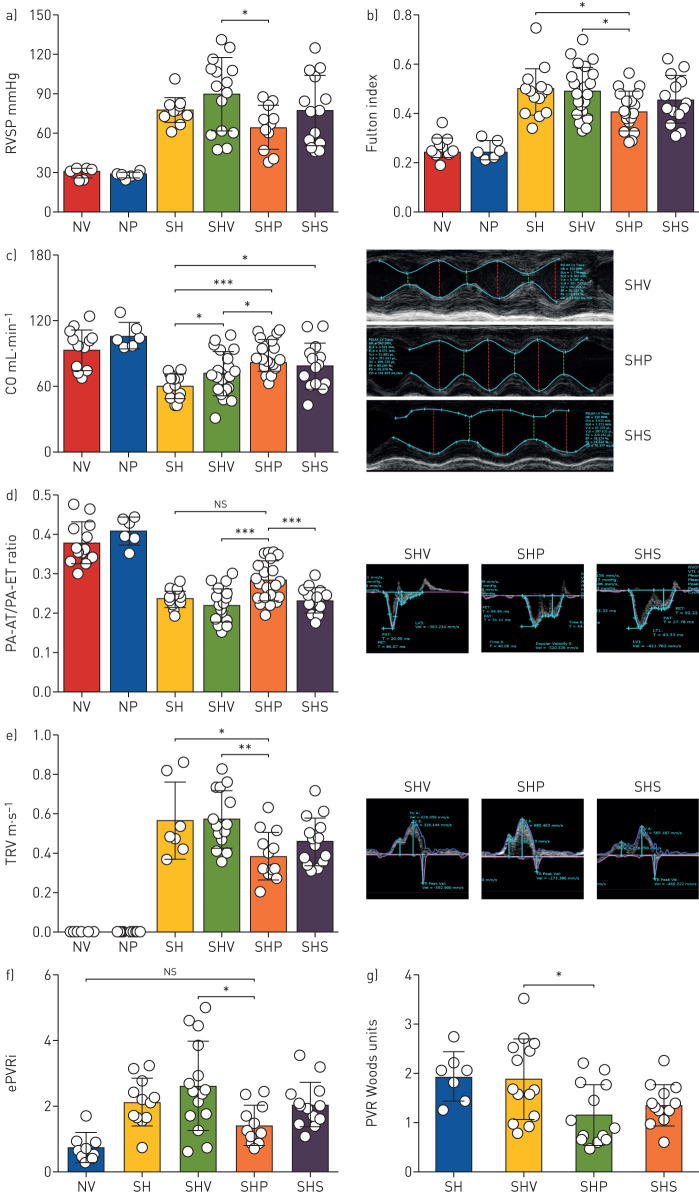
Hypoxia-inducible factor (HIF)2α-inhibitor decreases pulmonary vascular resistance (PVR) in established rat Su5416/Hx pulmonary hypertension (PH) model. Assessment of a) right ventricular systolic pressure (RVSP) and b) right ventricle hypertrophy (RVH; Fulton index); c) cardiac output (CO) in Sprague Dawley rats. Cardiac echo analysis of d) pulmonary acceleration time (PA-AT)/ right-ventricular ejection time (PA-ET) ratio with representative photomicrographs; e) tricuspid valve regurgitation (TVR)-velocity with representative photomicrographs for vehicle control, PT2567 or sildenafil. Calculation of pulmonary vascular resistance f) estimation-PVR index; g) Woods units. Data are presented as mean±sd. NV: normoxia vehicle (n=8); NP: normoxia/PT2567; SH: Su/Hx-3w (n=12); SHV: Su/Hx-vehicle (n=15); SHP: Su/Hx-PT2567 (n=15); SHS: Su/Hx-sildenafil (n=14). *: p<0.05, **: p<0.001, ***: p<0.0001 (one-way ANOVA).

We next evaluated whether an intervention strategy with PT2567 (100 mg·kg^−1^) would influence pulmonary vascular function and PH disease progression in su5416/hypoxia rats. As illustrated in supplementary figure S5a, all rats received the same su5416/hypoxia initiation process as described in the prevention protocol. Haemodynamic baseline was assessed 24 h after removal from the hypoxic chamber. These data provide a point of reference of disease severity for the intervention arms of the study. The remaining rats were randomly assigned to three groups for treatment with vehicle, PT2567 (100 mg·kg^−1^) or sildenafil (30 mg·kg^−1^) by oral gavage twice daily for 3 weeks. Normoxic control groups received vehicle or PT2567 treatment for 3 weeks. Haemodynamic analysis of the intervention arm was then completed 16–18 h after the final oral gavage. We believe this time delay facilitates the assessment of pulmonary vascular stasis rather than the vasotensive modulating properties of the compounds. We report that RVSP in the PT2567 group (64.85±16.86 mmHg, p<0.05) was significantly lower than the vehicle control group (89.82±27.92 mmHg) (figure 2a). Sildenafil (77.29±26.56 mmHg) did not lower RVSP when compared to vehicle-treated rats. In addition, the Fulton index (RVH) was significantly lower in the PT2567-treated group (0.4074±0.079) compared to vehicle alone (04906±0.098) (figure 2b); a strong correlation was noted between RVSP and RVH (r^2^=0.7066, p<0.0001) (supplementary figure S5b). Notably, PT2567 treatment significantly influenced the restoration of cardiac output (figure 2c) when compared to disease control or vehicle-treated PH rats. Cardiac echo analysis of PA-AT/PA-ET ratio (figure 2d) and tricuspid valve regurgitation (TVR) (figure 2e) suggest a reduction of pulmonary artery back-pressure with the potential to restore diastolic function. We analysed the mid-systolic decrease in PA flow velocity in the PA flow-time curves, known as the “notch” in rat models of PH. Notch duration has previously been reported and linked to PH disease severity [32]. We report a correlation between notch duration and pulmonary arterial pressure (estimated PAP) (supplementary figure 5c) with PT2567 intervention decreasing notch duration when compared to vehicle treated rats (supplementary figure 5d-e).

PVR is coupled to both pulmonary pressure and cardiac output and is thought to better reflect the pathophysiology of PH, as, to a large extent, it is a result of vascular remodelling in peripheral vessels. PVR was calculated by both catheterisation (estimated PVR index=estimated PAP−end-diastolic pressure/cardiac index) [33] and cardiac echo (Woods index(WU)=10×TVR/RVOT−VTI) (additional parameters used in PVR calculation are shown in supplementary figure S5f–i). PT2567 intervention significantly reduced the estimated PVR index (1.486±0.59, p<0.05) and Woods index (1.167±0.615, p<0.05) when compared to vehicle control (2.605±1.370 and 1.897±0.783, respectively) (figure 2f and g). HIF2α inhibition also restored RVOT-VTI to values similar to normoxic vehicle controls (supplementary figure S5j), while all other su5416/hypoxia-treated groups maintained substantially lower RVOT-VTI. Left heart blood pressure and heart rate were unaffected by either PT2567 or sildenafil when compared to normoxic vehicle control (supplementary figure S5k–l). Of note, normoxic-vehicle and normoxic-PT2567 groups did not show any difference across all haemodynamic analyses.

### PT2567 intervention reduces pulmonary vascular remodelling

**FIGURE 3:**
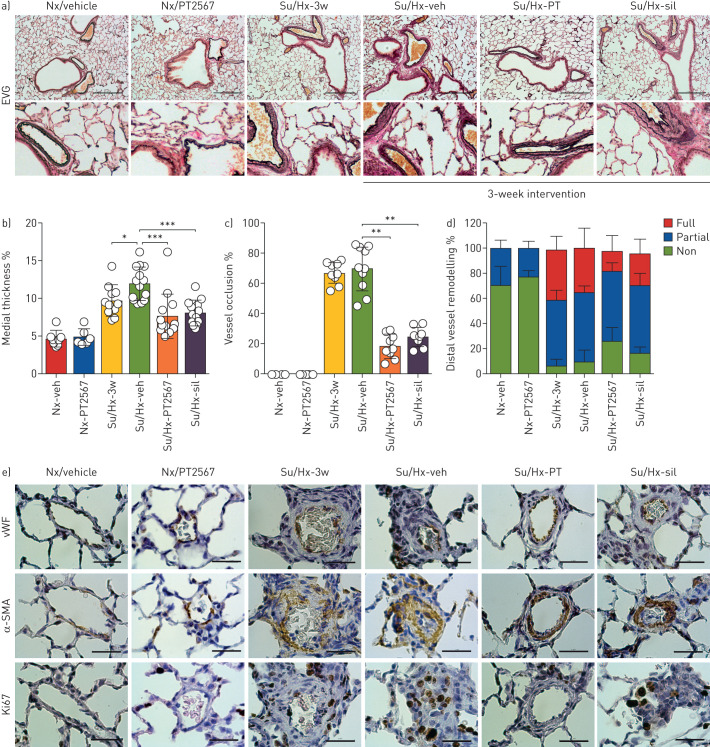
PT2567 decreases pulmonary vascular remodelling. a) Representative photomicrographs of lung sections stained with elastic van Gieson (EVG) (scale bar=0.30 mm); b) assessment of pulmonary artery wall thickness as a percentage of luminal diameter; c) determination of small arterial vessel (>50 μm) occlusion, shown as percentage fully occluded; d) quantification of non-, partially and fully muscularised arteries as a percentage of total alveolar wall and duct arteries; e) representative photomicrographs of lung sections immunostained for von Willebrand factor (vWF), α-smooth muscle actin (SMA) and Ki67 (scale bar=0.30 mm). Data are presented as mean±sd. Nx-veh (n=8), Nx/PT2567 (n=6), Su/Hx-3w (n=12), Su/Hx-veh (n=15), Su/Hx-PT2567 (n=11), Su/Hx-sil (n=14). Nx: normoxia; veh: vehicle; Su/Hx: su5416/hypoxia; sil: sildenafil. *: p<0.05, **: p<0.001, ***: p<0.0001 (one-way ANOVA).

We evaluated serial histological sections of inflated lungs for vascular remodelling. Lung sections were stained with EVG (figure 3a), von Willebrand factor and immunostained for α-smooth muscle actin (SMA), Ki67 (cell proliferation marker) (figure 3e) and MPO (supplementary figure S6g–h). Pulmonary histopathology scoring is presented in supplementary table S1b. The incidence of smooth muscle hypertrophy, perivascular/vascular inflammation and perivascular fibrosis was decreased in both PT2567 and sildenafil intervention groups when compared to su5416/hypoxia-vehicle group. Group mean scores for smooth muscle hypertrophy, perivascular/vascular inflammation and fibrosis were 0.66, 5.70, 8.90, 4.70 and 5.90 in normoxia vehicle, su5416/hypoxia, su5416/hypoxia-vehicle, su5416/hypoxia-PT2567 100 mg·kg^−1^ and su5416/hypoxia-sildenafil, respectively.

We assessed medial thickening in the larger bronchial associated pulmonary vessels as a measure of neointimal lesions. Rats exposed to su5416/hypoxia for 3 weeks showed a significant increase in medial thickness compared to normoxic vehicle controls with further remodelling noted in su5416/hypoxia-vehicle (figure 3b). In comparison, both PT2567 and sildenafil intervention demonstrated a significant reduction in medial thickness relative to su5416/hypoxia-vehicle (figure 3b). Next, we assessed the extent of small arterial occlusion. Vessels (≤50 μm) were determined to be either fully occluded or with an open lumen. PT2567 and sildenafil intervention significantly reduced the degree of vessel occlusion when compared to su5416/hypoxia or su5416/hypoxia-vehicle (figure 3c). Furthermore, lung sections from su5416/hypoxia-vehicle showed an increase in elastin and α-SMA associated with pulmonary vessels (figure 3e, supplementary figure S6a). This was partially inhibited by PT2567 intervention, with a substantial reduction in α-SMA deposition in distal vessels, particularly of fully muscularised vessels compared to the su5416/hypoxia-vehicle group (figure 3d, supplementary figure S6b–d). Quantification of the expression of markers of proliferation (Ki67) further confirmed that both PT2567 and sildenafil reduced su5426/hypoxia-induced pulmonary vessel proliferation (supplementary figure S6e) and, more specifically, PAEC proliferation (supplementary figure S6f). Finally, quantification of MPO in lung sections revealed reduced immunoreactivity in perivascular regions of rats treated with PT2567 when compared to su5426/hypoxia-3w and su5426/hypoxia-vehicle animals, with no change in MPO immunoreactivity in intravascular regions (supplementary figure S6g,h).

Taken together, these data suggest that inhibition of HIF2α function with PT2567 reduces pulmonary vascular remodelling and the dynamic changes in cardiovascular function associated with this PH model.

### PT2567 modulates PH-associated gene expression and normalises plasma nitrite levels

Having identified a difference in pulmonary vascular remodelling with PT2567, we next investigated the pulmonary expression profiles of known HIFα target genes associated with glycolysis, inflammation and pulmonary remodelling in whole-lung samples. Relatively little is known about the global inhibition of HIF2α and effects on the multicellular componentry of the lung in PH models. Given the systemic administration of PT2567 it was thought advantageous to analyse the whole lung. PT2567 intervention normalised the expression of known HIF2α target genes, *glut1* and *ca9* when compared to su5416/hypoxia-vehicle control (supplementary figure S7a,b), with little effect on HIF1α target gene expression, *ldha* and *pgk1* (supplementary figure S7c,d). Su5416/hypoxia significantly increased the gene expression of inflammatory targets *cxcl12* and receptor *cxcr4*, and endothelial adhesion molecules *icam1* and *sele.* PT2567 intervention reduced gene expression to near normoxia nondisease animals (supplementary figure S7e–h). Next, we analysed signalling targets associated with PH: *apln*, *arg2*, cell-cycle *ccnd1*, *pai1* and vasoactive *edn-1* were all reduced and *id1* expression restored following PT2567 intervention (supplementary figure S7i–n). Sildenafil normalised the expression of *glut1*, *cxcl12* and *id1*, but did not impact on the expression of any other genes.

Given that PAH pathophysiology increases RVH which often ends in right-heart failure and death, we next investigated the expression of structural and stress-associated genes in RV tissues. PT2567 significantly reduced the expression of myosin heavy chain-7 (*myh7*) and actin-alpha-1 (*acta1*) and myosin light chain-3 (*myl3*) trended towards normal expression (p=0.065 nonparametric analysis) when compared with su5416/hypoxia-vehicle treated rats (supplementary figure S8a–c). The cardiac stress genes natriuretic peptide A (*nppa*), natriuretic peptide B (*nppb*) and annexin A5 (*anxa5*) and the fibrosis targets collagen type-1 A1 (*col1a1*) type-3 a1 (*col3a1*) and tissue inhibitor of metalloproteinase 2 (*timp2*) were reduced following PT2567 intervention (supplementary figure S8d–i); sildenafil only reduced the expression of *col3a1* and *timp2*.

**FIGURE 4:**
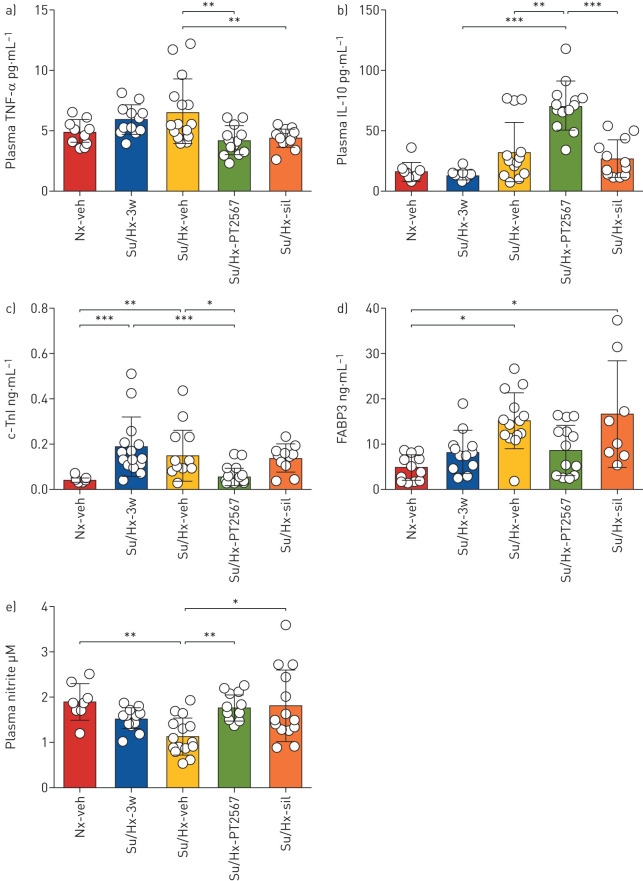
Hypoxia-inducible factor (HIF)2α-inhibition restores plasma nitrite levels. a–d) Plasma cytokines and cardiac stress factor analysis a) tumour necrosis factor (TNF)-α, b) interleukin (IL)-10, (Nx-veh n=8, Su/Hx n=12, Su/Hx-veh n=15, Su/Hx-PT2567 n=11, Su/Hx-sildenafil n=14). c) c-Troponin-I (c-TnI), d) fatty acid binding protein-3 (FABP3) (Nx/Veh n=6, Su/Hx-3w n=6, Su/Hx-veh n=8, Su/Hx-PT2567 n=8, Su/Hx-sildenafil n=8). e) Plasma nitrite assessed by nitric oxide analyser (Sievers) (Nx/veh n=8, Su/Hx-3w n=12, Su/Hx-veh n=15, Su/Hx-PT2567 n=11, Su/Hx-sildenafil n=14). Data are presented as mean±sd. Nx: normoxia; veh: vehicle; Su/Hx: su5416/hypoxia; sil: sildenafil. *: p<0.05, **: p<0.001, ***: p<0.0001 (one-way ANOVA).

Next, we analysed plasma samples for cytokines, markers of cardiac stress and nitrites in the su5416/hypoxia rat model. PT2567 intervention significantly reduced plasma tumour necrosis factor (TNF)-α concentration (figure 4a) and increased immunosuppressive interleukin (IL)-10 (figure 4b) when compared to su5416/hypoxia-vehicle rats. Other plasma cytokines including IL-13, IL-4, IL-5, interferon-γ, IL-1β and IL-8 were also measured (table 2), but did not reach significance when compared to su5416/hypoxia-vehicle control.

**TABLE 2:** Plasma cytokine analysis

	**IFN-γ pg·mL^−1^**	**IL-5 pg·mL^−1^**	**IL-1β LOD 12 pg·mL^−1^**	**IL-8 pg·mL^−1^**	**IL-4 LOD 2 pg·mL^−1^**	**IL-13 pg·mL^−1^**
Nx-veh	4.45±0.68	19.26±2.61	12.31±0.62	40.70±5.57	3.83±0.88	4.53±0.23
Su/Hx 3-weeks	5.28±1.70	24.55±4.37	12.60±0.42	44.83±5.66	3.36±0.89	5.67±0.91
Su/Hx-veh	6.43±0.96	26.77±4.90	14.05±0.91	51.19±4.18	3.68±0.53	6.45±0.75
Su/Hx-PT2567	9.90±1.62	21.44±2.46	12.49±0.47	48.44±4.30	5.21±0.79*	8.46±0.94*
Su/Hx-sil	5.36±1.10	25.42±2.65	12.41±0.31	53.95±7.82	2.26±0.20	4.35±0.15

Data are presented as mean±sd. IFN: interferon; IL: interleukin; LOD: limit of detection; Nx: normoxia; veh: vehicle; Su/Hx: su5416/hypoxia; sil: sildenafil. *: p<0.05 when compared with Nx-veh (one-way ANOVA).

We also noted the normalisation of plasma cardiac stress markers c-Troponin-I and fatty acid binding protein-3 in the PT2567-treated group when compared to su5416/hypoxia-vehicle (figure 4c,d).

Consistent with PH disease severity, we observed a significant decrease in plasma nitrites in su5416/hypoxia-vehicle (1.121±0.414 μM) when compared to normoxia-vehicle (1.889±0.404 μM). Notably, both PT2567 and sildenafil restored plasma nitrite concentrations to near normoxia-vehicle control levels (1.756±0.294 μM and 1.798±0.795 μM, respectively) (figure 4e).

### PT2567 modulated blood counts within normal physiological limits

Whole-blood analysis showed no change in white cell count. However, PT2567 treatment reduced red blood cell count, haemoglobin levels and percentage haematocrit towards the lower reference value within the physiological range. However, these readings were not significantly different to vehicle control (supplementary figure S9a–d).

### Carotid body size is not affected during PT2567 intervention

Recent studies have shown that HIF2α is essential for carotid body development and growth in response to chronic hypoxia [34, 35]. Given the importance of the carotid body in regulating cardiorespiratory responses to hypoxia, we sought to determine whether PT2567 treatment impacted carotid body morphology and by extension its function. Histological analysis of carotid bifurcations from rats exposed to su5416/hypoxia for 3 weeks showed a significant increase in carotid body volume compared to Nx-control rats (supplementary figure S10a,b,f). Typical carotid body shrinking across the re-oxygenation period was unaffected in both PT2567 or sildenafil-treated rats compared to vehicle control group (supplementary figure S10c–f).

### PT2567 intervention increases survival rate in MCT-challenged rats

In addition, we investigated the effect of PT2567 intervention in the MCT rat model of PH. As illustrated in supplementary figure S11a, 14 days after MCT challenge the rats received intervention with vehicle, PT2567 (100 mg·kg^−1^) or sildenafil (30 mg·kg^−1^) for 2 weeks. Cardiac echo was recorded 14 days following MCT challenge and at the end-point of the intervention study (28 days). PT2567 intervention resulted in a 90% survival rate compared to 77% for sildenafil-treated animals (supplementary figure S11b). Cardiac echo showed that PT2567 intervention offered a degree of protection for PA-AT/PA-ET (ratio), RVOT-VTI and cardiac output (supplementary figure S11c–e) when compared to vehicle-treated animals. Sildenafil-treated animals were not significantly different to vehicle-treated animals across all cardiac echo parameters.

### Inhibition of HIF2α normalises PAH patient-derived BOEC proliferation and arginase activity

BOECs have been extensively characterised and used as a model for studying *in vitro* endothelial function in vascular disorders [36, 37]. The BOECs used in this study were previously characterised by flow cytometry immunostaining for CD133, CD34, VEGFR2 [36] and gene-array analysis that demonstrated their close functional and gene expression similarity to PAECs [38]. Additionally, we previously demonstrated that these cells had no deficiencies in HIFα expression or hypoxic induced stability of these transcription factors [16].

First, we evaluated the activity and specificity of PT2567 (1 μM) in the aforementioned BOECs isolated from PAH patients and healthy volunteers. Human qPCR primers were selected to complement the analysis of rat whole lung gene expression used in the su5416/hypoxia model.

**FIGURE 5:**
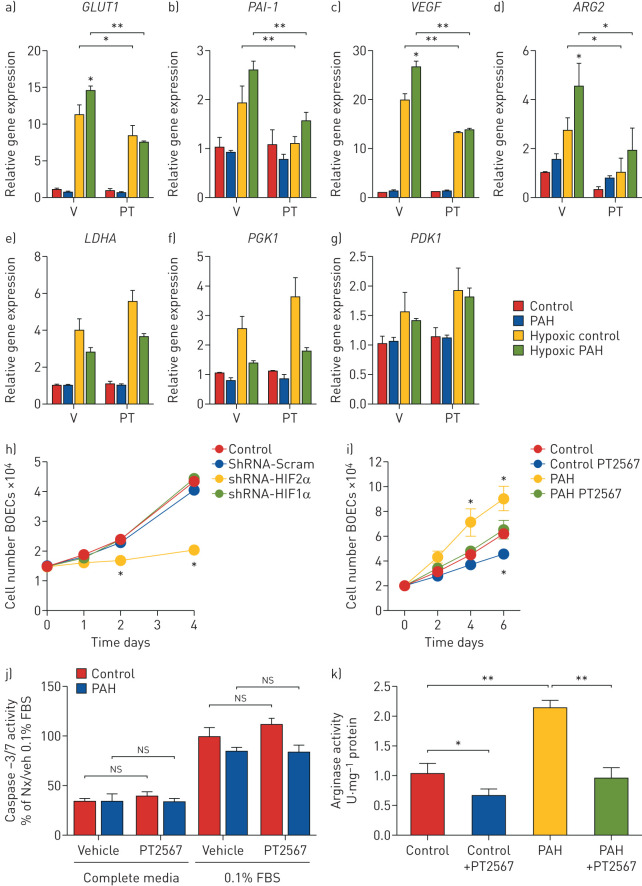
Analysis of human blood outgrowth endothelial cells (BOECs) as a model for studying *in vitro* endothelial function in pulmonary arterial hypertension (PAH). a–g) Human BOECs were exposed to hypoxia +/− PT2567 (PT) or vehicle (V), gene expression determined by quantitative PCR: a) *GLUT1*, b) *PAI-1*, c) *VEGF*, d) *ARG2*, e) *LDHA*, f) *PGK1*, g) *PDK1*. h) Control BOEC proliferation following hypoxia-inducible factor (HIF)α knockdown. i) Control and PAH BOEC proliferation +/− PT2567. j) BOEC apoptosis determined by caspase-3/7 activity. BOECs were cultured in complete endothelial media or basal endothelial media with 0.1%-fetal bovine serum (FBS) +/− PT2567 for 18 h. Data corrected to positive control (0.1% FBS control BOECs with vehicle) percentage mean activity±sem. k) Arginase activity assay from control and PAH BOEC +/− PT2567. Human BOECs from healthy volunteers (n=3) or PAH patients (n=3); data are presented as mean±sem. *: p<0.05, **: p<0.001 (one-way ANOVA).

Hypoxic exposure of BOECs from healthy volunteers and PAH patients increased both HIF1α and HIF2α target genes (figure 5a–g) consistent with our previous study. We also reported that hypoxia induced increase in *GLUT1*, *VEGF* and *ARG2* expression were greater in PAH BOECs when compared to control BOECs. Treatment with PT2567 markedly reduced hypoxia-induced *GLUT1*, *PAI-1*, *VEGF* and *ARG2* expression without effecting HIF1α target gene transcripts *LDHA*, *PGK1* and *PDK1* in both control and PAH BOECs (figure 5a–g).

Hyperproliferation is a documented phenotype of BOECs isolated from PAH patient [36]. We initially utilised a short-hairpin knockdown strategy for HIF1α or HIF2α in control BOECs to initially assess the impact of these transcription factors on proliferation. shRNA-HIF2α substantially reduced cell proliferation in control BOECs on days 2–4 when compared to shRNA-Scramble, whereas shRNA-HIF1α had little/no effect on proliferation (figure 5h). We next investigated the effect of PT2567 on BOEC proliferation. As previously documented, BOECs from PAH patients demonstrated a considerably higher rate of proliferation by day 4 and maintained to day 6 when compared to BOECs from healthy volunteers. Treatment with PT2567 reduced cell proliferation from both PAH patient and healthy volunteer derived BOECs (figure 5i) without affecting the rate of cellular apoptosis as determined by caspase-3/7 activity (figure 5j).

We have previously documented the aberrant expression and activity of arginase-2 in BOECs isolated from PAH patients [16] (human PAECs do not express arginase-1). Treatment with PT2567 for 24 h suppressed arginase-2 enzyme activity in BOEC from PAH patients and healthy volunteers (figure 5k). Taken together, these data demonstrate that targeting of HIF2α in BOECs derived from PAH patients, reduced aberrant HIF2α target genes expression and reduced the hyperproliferative endothelial phenotype and arginase-2 activity to near healthy controls. Finally, we assessed the impact of PT2567 on BOECs network formation function. BOECs networks from PAH patients have been documented to be more fragile with shorter connections and smaller loops when compared to controls [36]. This network phenotype is partially corrected following PT2567 treatment by increasing both tube length (supplementary figure S11a) and loop size (supplementary figure S11b) to values similar as control BOECs. Representative photomicrographs of BOEC network formation following vehicle or PT2567 (1 μM) treatment in healthy control BOEC (supplementary figure S11c) and PAH BOEC (supplementary figure S11d) are shown.

## Discussion

In this study we have shown that HIF2α signalling is a key element in the development of PH and that specifically targeting this process with a novel global HIF2α inhibitor (PT2567) during the initiation phase (figure 1) or as an intervention strategy (figure 2) alleviated many of the pathologies associated with PH disease.

The HIF2α inhibitor PT2567, used throughout this study, binds directly to a region within the PAS-B domain of HIF2α and disrupts the formation of HIF2α/ARNT dimer, a key event essential for HIF2α activation. Our *in vitro* studies confirmed the high affinity and specificity of PT2567 for HIF2α and the low micromolar concentration required for HIF2α target gene inhibition. In addition, we report that HIF2α signalling is potentially involved in the hyperproliferative and endothelial network phenotype observed in BOECs derived from PAH spatient *via* the expression of HIF2α target genes (*GLUT1*, *VEGF*, *PAI2* and *ARG2*).

Our *in vivo* pharmokinetic analysis identified a favourable oral bioavailability and plasma stability profile in adult rodents (supplementary figures S1–S4) at concentrations subsequently used in both prevention and intervention protocols. The su5416/hypoxia prevention protocol confirmed several key observations previously described in murine HIF2α manipulation models of PH, but also further established that inhibition of global HIF2α activity directly influenced PH disease initiation. PT2567 intervention reduced pulmonary vascular resistance and vascular remodelling and increased cardiac function in the su5416/hypoxia model, and offered a significant survival advantage in the MCT PH model. Sildenafil was used as a clinical comparative throughout these studies, but offered only a small beneficial effect in both prevention and intervention studies. We think this discrepancy is probably due to the timing of the haemodynamic analysis, being completed 16–18 h after the final dose, and therefore potentially outside the vasoactive window.

Although increased HIF2α protein stability has been identified in pulmonary vascular occlusive lesions from both idiopathic PAH patients and rodent PH models, the mechanistic role of HIF2α in the initiation and progression of PH remains unclear. The vast majority of published *in vivo* studies that question a role for HIFα in the initiation and development of PH use tissue-specific gene deletion mouse models. Although these tissue-specific mouse models are incredibly useful to determine gene contribution in disease development, they do not take account of potential developmental shortcomings that may influence normal physiological or pathological responses, and how specific gene function may differ across multiple vascular beds to influence disease progression or severity.

The primary goal of this study was to determine the impact of global HIF2α inhibition in preventing and reverting pulmonary hypertension and to investigate the pathological influence of this transcription factor in regulating the expression of potential PH associated target genes that systematically influence vascular homeostasis.

To this end, we investigated the gene expression profiles in whole-lung samples from our rodent PH models to ensure the inclusion of all pulmonary cell types necessary for PH disease progression. These data confirmed the specificity of PT2567, and showed reduced expression of inflammatory (*cxcl12*, *cxcr4*, *icam1*, *sele*) and signalling targets (*apln*, *arg2*, *ccnd1*, *pai1*, *edn1*) associated with PH with the additional restoration of *id1* gene expression. Although the impact of sildenafil treatment on haemodynamic parameters appeared less impressive in these animals studied, the influence of sildenafil on whole-lung gene expression patterns was not dissimilar to PT2567. Further studies with concomitant or alternate PT2567 and sildenafil treatment may be clinically relevant.

Recent studies by Dai *et al.* [39] and Hu *et al.* [40] offer further support for the important role of HIF2α in PH and confirmed many of these inflammatory and signalling target genes. Furthermore, Hu *et al.* [40] utilised PT2567 in a hypobaric hypoxic rat model of PH and reported a reduction in both pulmonary arterial pressures and vascular remodelling. In line with this, our study confirmed a reduction in pulmonary vascular smooth muscle deposition and shows a reduction in pulmonary vascular endothelial cell proliferation and MPO-positive cells (myeloid-lineage) in the perivascular regions.

These finding are consistent with those previously published using murine tissue-type specific HIF2α deletion models, reporting a reduction in endothelial vasoactive function (*edn1*, *arg-2*, *apln*) [8, 9, 16], cellular proliferation/angiogenesis (*ccnd1*, *vegf*) [16] and endothelial-to-mesenchymal transition (*snai1/2*) targets [41].

We previously documented that deletion of pulmonary endothelial HIF2α reduced circulating pro-inflammatory cytokines and growth factors (IL-6, IL-8, TNF-α, IL-1β, Cxcl12) in the chronic-hypoxia PH model [16]. High circulating concentrations of these factors are associated with poor PAH patient prognosis [42]. We report that PT2567 intervention reduced circulating TNF-α and increased immune-suppressive IL-10 plasma concentrations. A previous report described the administration of recombinant IL-10 to MCT-challenged rats reduced haemodynamics and pulmonary vascular remodelling and provided a survival advantage over vehicle control [43]. Other murine inflammatory models have linked aberrant myeloid HIF2α expression with low plasma IL-10 concentrations, where mice deficient in myeloid-HIF2α restored/increased plasma IL-10 [44]. Further investigations would be required to clarify the cellular source (regulatory T-cells, macrophages, B-cells, dendritic cells) and role of HIFα modulation of IL-10 in PAH.

The circulating concentration of nitric oxide (NO) is key to the structural integrity and function of the cardiovascular system. Reduced NO bioavailability is associated with endothelial dysfunction, smooth muscle proliferation and with implications in the development of cardiovascular and PAH disease severity [45–48]. We show here that plasma NO/nitrite concentrations are restored to near normal/normoxia values following intervention with PT2567 or sildenafil. These data are consistent with those of our previous investigations in the murine chronic-hypoxia PH model [16], where we described a HIF2α–Arg2 axis as essential for the development of PH. We showed that increased arginase expression and activity led to dysregulation of vascular NO homeostasis [16]. Sildenafil indirectly modulates endothelial function *via* endothelial nitric oxide synthesis [49] in models of PH. However, the action of sildenafil on plasma nitrite is thought to be independent of HIF signalling

Given the global HIF2α inhibition of PT2567, other nonpulmonary tissues may also indirectly modulate PH disease progression. We report that PT2567 intervention reduces the expression of several RV genes associated with structural and stress-induced adaptation; however, based on what little is known about HIF2α signalling in cardiovascular disease we cannot fully differentiate restoration of cardiac function as a direct effect of PT2567 or indirectly as a result of pulmonary vascular function.

The role of HIF1α in the initiation and development of PH remains controversial. The lack of specific HIF1α inhibitors hampers potential intervention studies in the rat su5416/hypoxia or MCT models. Therefore, the vast majority of the studies used tissue-specific knockout mouse models that demonstrated variable outcomes depending on the tissues targeted and the methodology used to aberrantly activate (PHD or Von Hippel–Lindau) or inhibit HIF1α stability. To some extent, targeting HIF1α signalling in smooth muscle cells has shown a beneficial effect in slowing down the progression of the disease [50]. We previously investigated the role of pulmonary endothelial HIF1α in a chronic hypoxia murine model of PH. We did not observe a difference in PH disease development over a 7–14–21-day hypoxic time course when compared to wild-type animals. Furthermore, Hu *et al.* [40] recently reported that global inhibition of HIF1α in a chronic hypoxic model of PH did not support a central role for this transcription factor in PH development. Further investigations are required to clarify if HIF1α or the combination of HIF1α/HIF2α influences the development of PH.

The current consensus view supports a role for HIF2α in the development of PAH, most probably through influencing multiple signalling pathways across pulmonary vascular beds leading to the progressive loss of vascular identity/plasticity/homeostasis that drives remodelling and increases vessel resistance. The degree of protection offered by PT2567 intervention in this article may be attributed, to some extent, to the modulation of multiple PH associated target genes for *glut1*, *cxcl12*, *apln*, *edn1*, *icam1*, *sele* and *ccnd1* in pulmonary tissues, although contributions from other known PAH targets including *bmpr2* [26], *il-6* [51], *sox17* [52] and *cav1* [53] across multiple tissues and other organs are most probable and cannot be ruled out.

The roles played by aberrant HIF2α stability and function in the development of PH has been an area of intense interest for nearly two decades [8, 9, 12, 16, 54, 55]. These collective studies clearly elucidate a pivotal role of vascular HIF2α in PH disease initiation and progression and support its evaluation as a novel therapeutic target for the treatment of PH patients. However, unlike the uniform genetic background of *in vivo* murine models used to establish genetic targets, PAH is a complex dynamic disease characterised by considerable patient heterogeneity. Thus, further investigations will be needed to determine the overall sensitivity of PAH population to HIF2α inhibitors and also clarify the combination effect of PT2567 with current therapeutic strategies. We believe the current organ-on-a-chip technology combined with BOECs from PAH patients could provide an excellent platform to further characterise the role of HIF2α in PH.

In conclusion, our work provides a greater understanding of direct inhibition of HIF2α transcriptional activity in PH initiation and progression in the su5416/hypoxia rodent model of PH. Further research is required to determine the position of HIF2α in the hierarchy of factors required to initiate PH and how prolonged HIF2α inhibition impacts not only on PH disease pathology but also in other essential physiological processes.

## Supplementary material

10.1183/13993003.02061-2019.Supp1 **Please note:** supplementary material is not edited by the Editorial Office, and is uploaded as it has been supplied by the author.
Supplementary figures ERJ-02061-2019_Supplementary_figures
Supplementary tables ERJ-02061-2019_Supplementary_tables

## Shareable PDF

10.1183/13993003.02061-2019.Shareable1 This one-page PDF can be shared freely online.
Shareable PDF ERJ-02061-2019.Shareable
