# Supplementary material for: Targeting HIF2α-ARNT hetero-dimerisation as a novel therapeutic strategy for pulmonary arterial hypertension
Source: Eur Respir J. 2021 Mar 4;57(3):1902061. doi: 10.1183/13993003.02061-2019 (PMC7930471; doi:10.1183/13993003.02061-2019)
Supplement: Supplementary file 1 [file ERJ-02061-2019_Supplementary_figures.pdf]

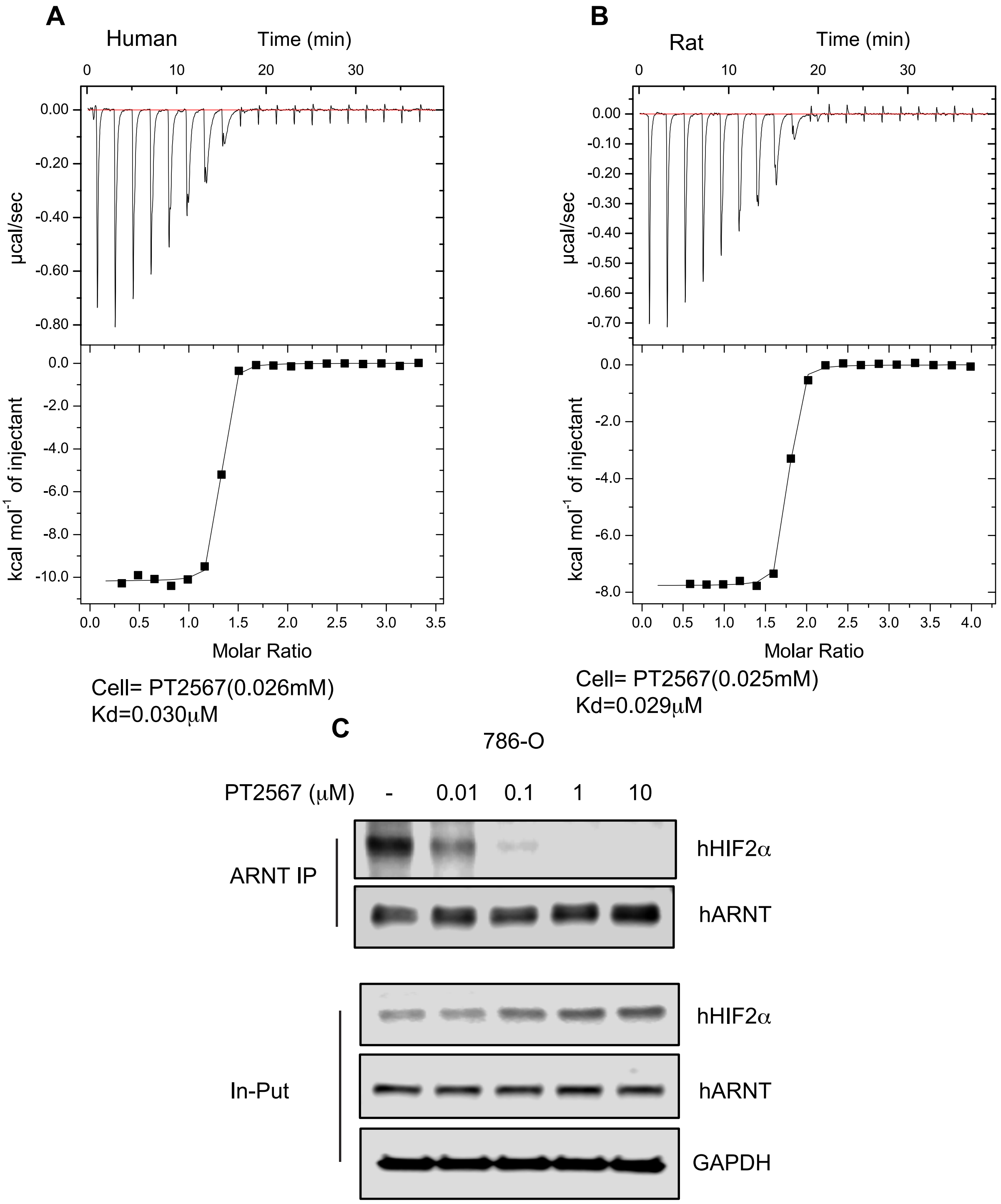

Figure S1. Binding of PT2567 to HIF2α PAS-B domain disrupts HIF2α/ARNT dimer formation.
A, B Binding affinity of PT2567 to PAS-B domain of (A) human HIF2α and (B) rat HIF2α using isothermal titration calorimetry.
C Co-immunoprecipitation experiment in 786-O cells demonstrating PT2567 disrupts HIF2α/ARNT dimerization

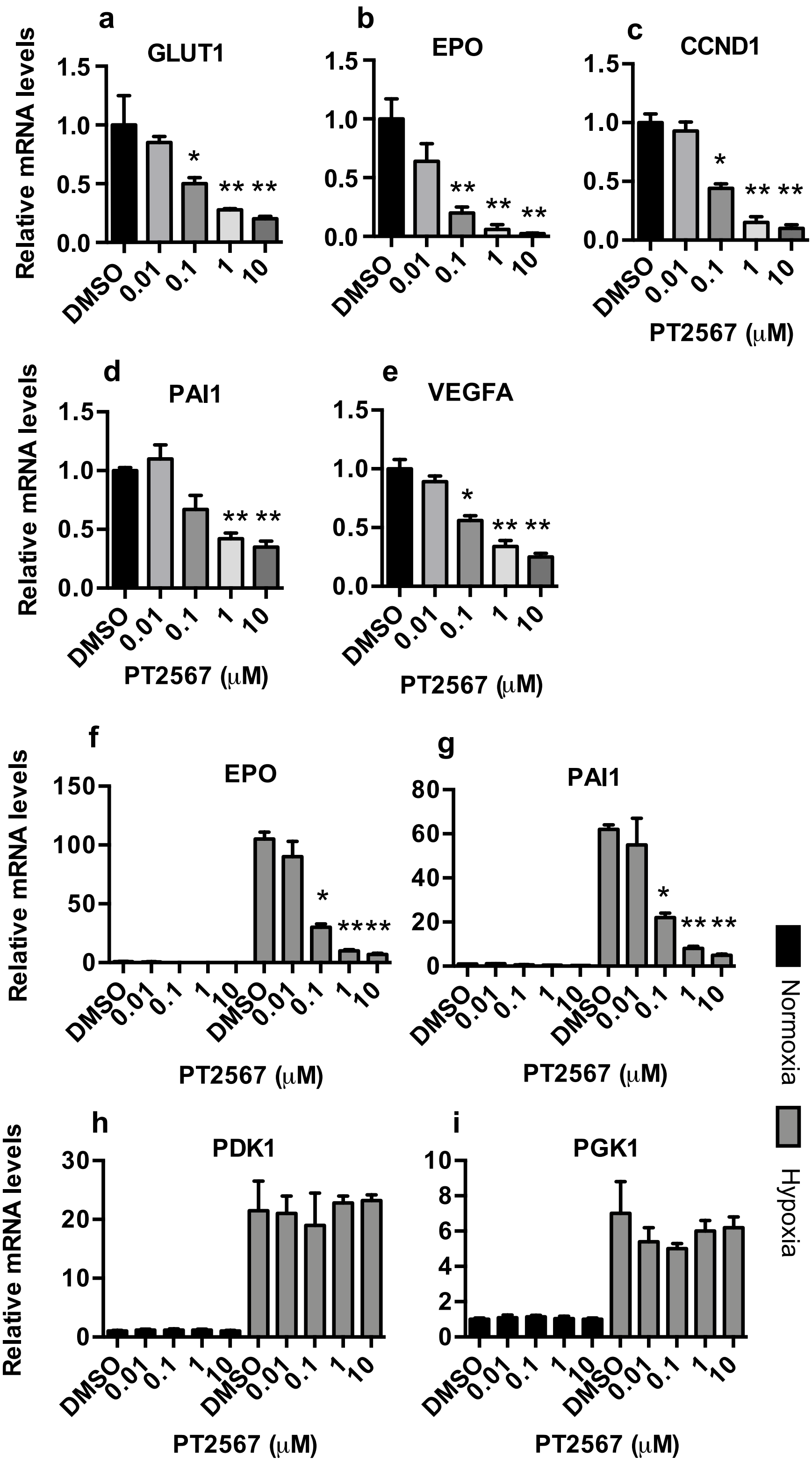

Figure S2. **PT2567 selectively inhibits HIF2α-target gene expression in 786-O and Hep3B cells.**
a-e PT2567 reduces 786-O mRNA levels for (a) GLUT1 (b) EPO, (c) CCDN1, (d) PAI1, and (e) VEGFA.
f-i PT2567 inhibits hypoxic stimulated HIF2α target gene expression (f) EPO, and (g) PAI1 without influencing HIF1α target genes (h) PDK1 and (i) PGK1 in Hep3B cells.
Data information mean ± SD (n=5) *p<0.05, **p<0.001 (one-way ANOVA)

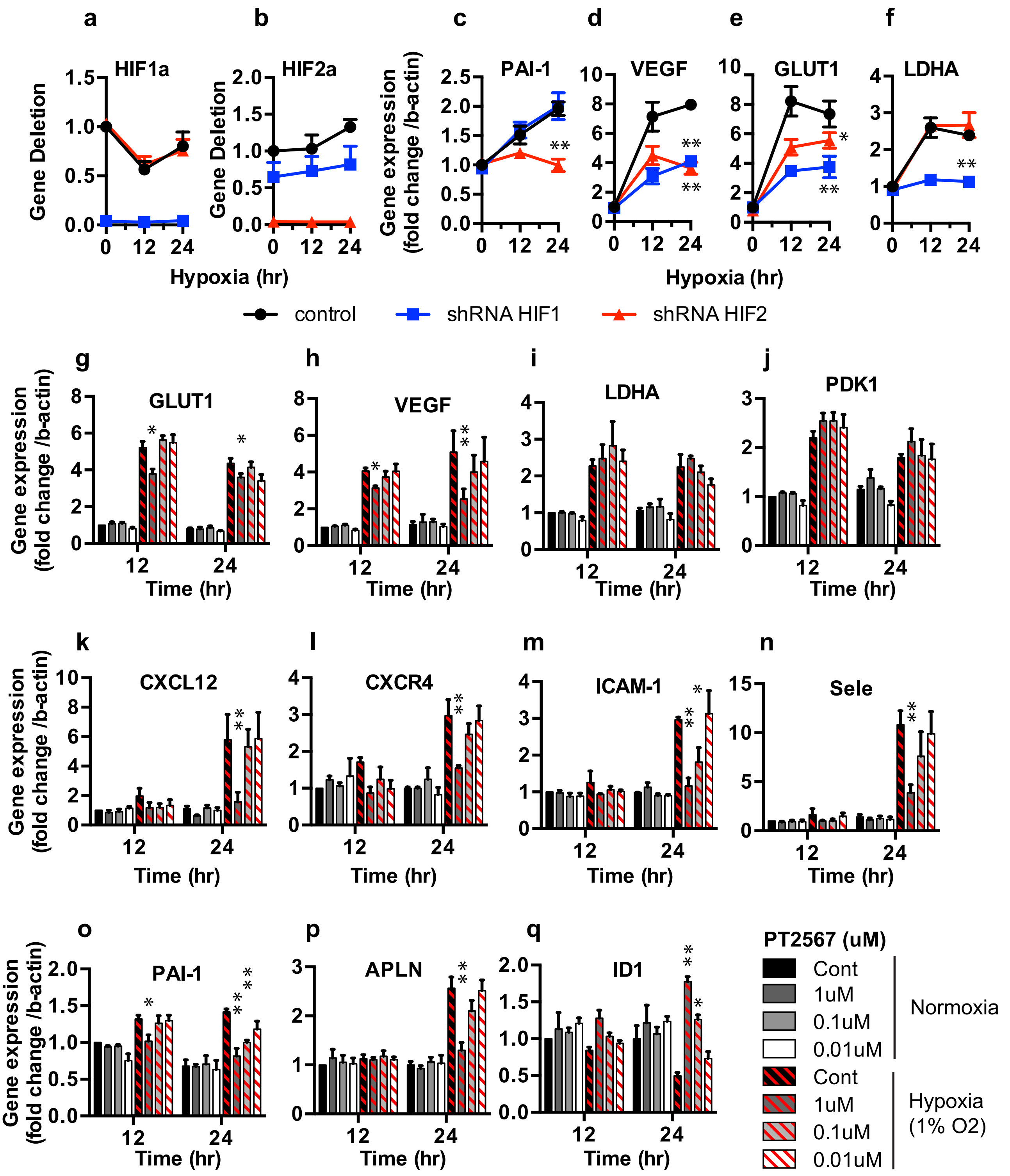

Figure S3. Validation of PT2567 specificity and activity in hPAEC.
a-f Human-PAECs were exposed to hypoxic time course +/- sh-RNA-HIF1α or –HIF2α, gene expression determined by qPCR (a) HIF1α, (b) HIF2α, (c) PAI-1, (d) VEGF, (e) GLUT1, (f) LDHA.
g-k PT2567 concentration response in hypoxia exposed hPAEC, gene expression determined by qPCR (g) GLUT1 (h) VEGF, (i) LDHA, (j) PDK1, (k) CXCL12, (l) CXCR4 (m) ICAM-1, (n) Sele, (o) PAI-1, (p) APLN, (q) ID1
Data information mean ± SEM (n=3) *p<0.05, **p<0.001 (T-Test)

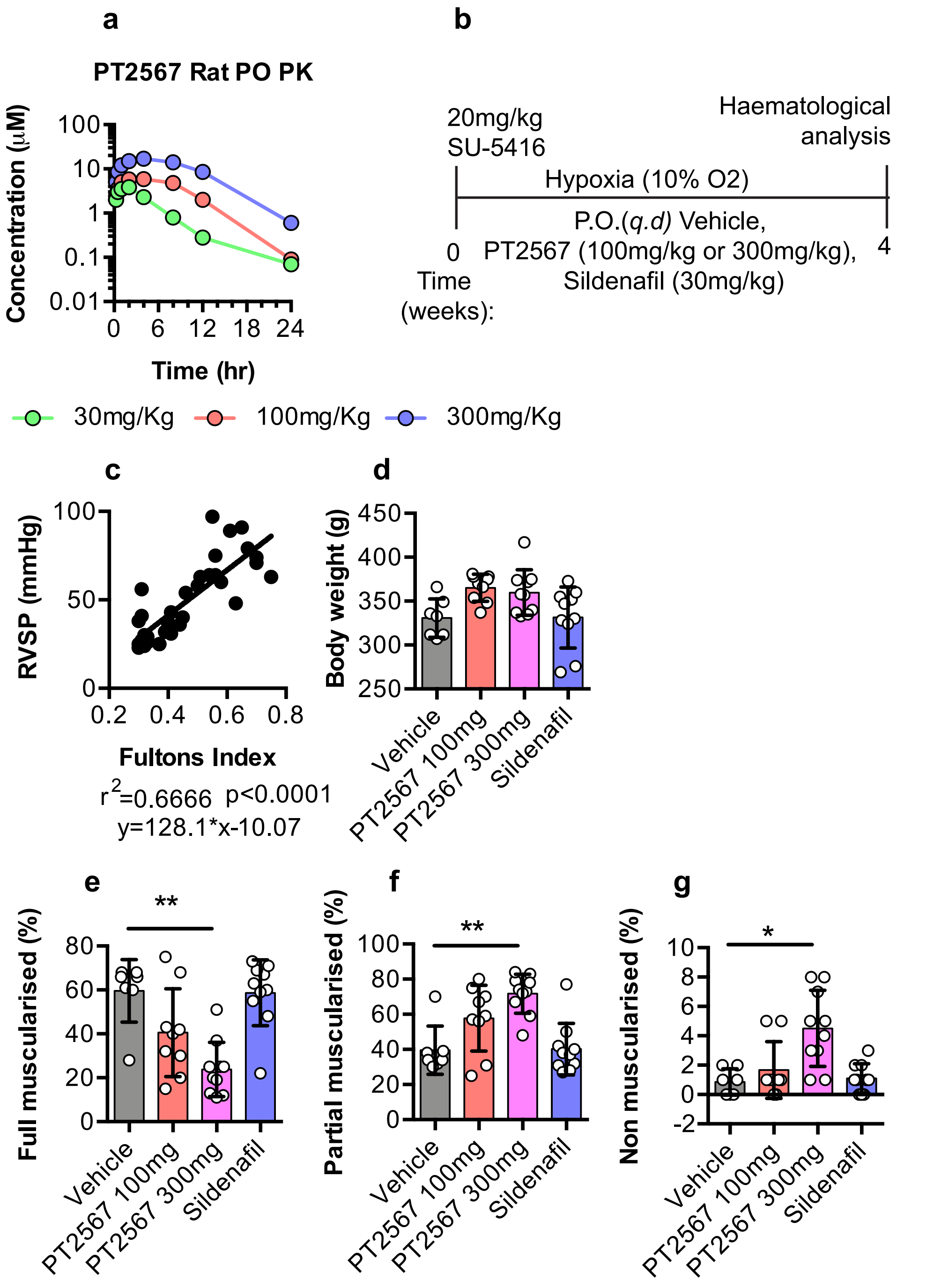

Figure S4. Rat PT2567 PO PK and PAH prevention profiles

a Plasma concentration versus time profiles of PT2567 after oral administration of 30, 100, 300mg/Kg.

b Line-diagram depicting the rat PH treatment strategy.

c Assessment of correlation between RVSP and RVH in the SU-5416 hypoxia prevention model (r2=0.666, y=128.1*x-10.07).

d Final body weight (g)

e-g Quantification of distal muscularisation process characterised as (e) fully muscularised, (f) partially muscularised or (g) non-muscularised.

Data Information mean ± SD (SuHx Veh n=8, SuHx PT2567 (100mg/kg) n=9 (300mg/kg) n=10, SuHx sildenafil n=10)*p<0.05, **p<0.001 (one-way ANOVA)

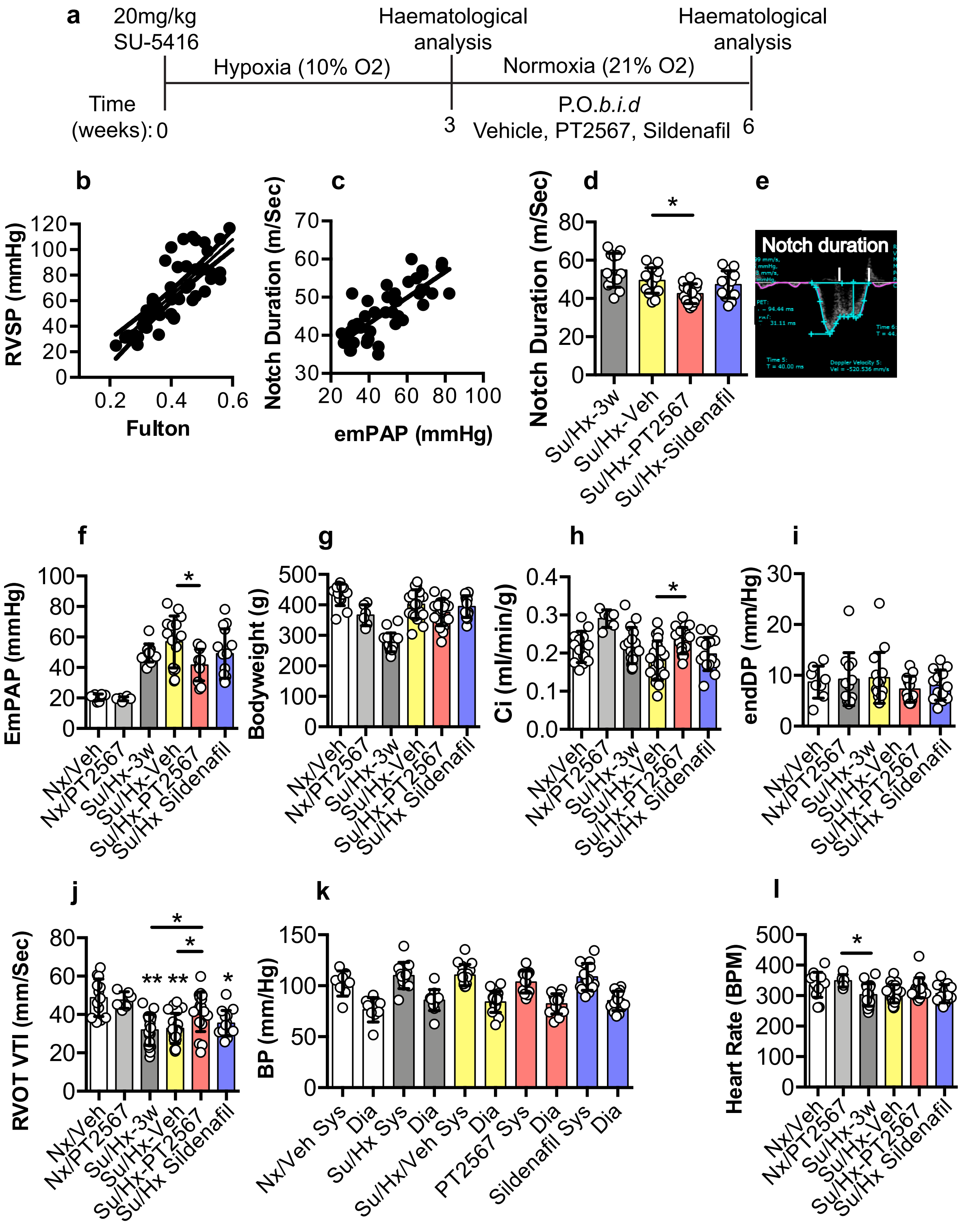

Figure S5 PT2567 restores PAT in a SU-5416/Hx intervention model of PH.
a Line-diagram depicting the rat PH treatment strategy
b, c Assessment of correlation between (b) RVSP and RVH in the SU-5416/Hx intervention model ($r2=0.7066$, $y=0.220.3x-24.25$, $p<0.0001$). (c) Notch duration and emPAP in the SU-5416/Hx intervention model ($r2=0.5944$, $y=0.3381*x29.58$).
d, e Cardiac echo was used to determine (d) Notch duration (e) Representative image showing Notch duration.
f-j Parameters used to calculate PVR (f) Estimation-PAP (mm/Hg) (g) Final body weight (g) (h) Cardiac-index (mmHg/g) (i) End diastolic pressure (mmHg) (j) RVOT VTI.
k Assessment of left ventricle function (mm/Hg) h, heart rate following intervention with vehicle, PT2567 or sildenafil, Data information mean ± SD Nx/Veh n=8-11, Nx/PT2567 n=6, SuHx-3w n=12-18, SuHx-Veh n=15-22, SuHx-PT2567 n=11-19, SuHx-sildenafil n=14. $*p<0.05$, $**p<0.001$ (one-way ANOVA)

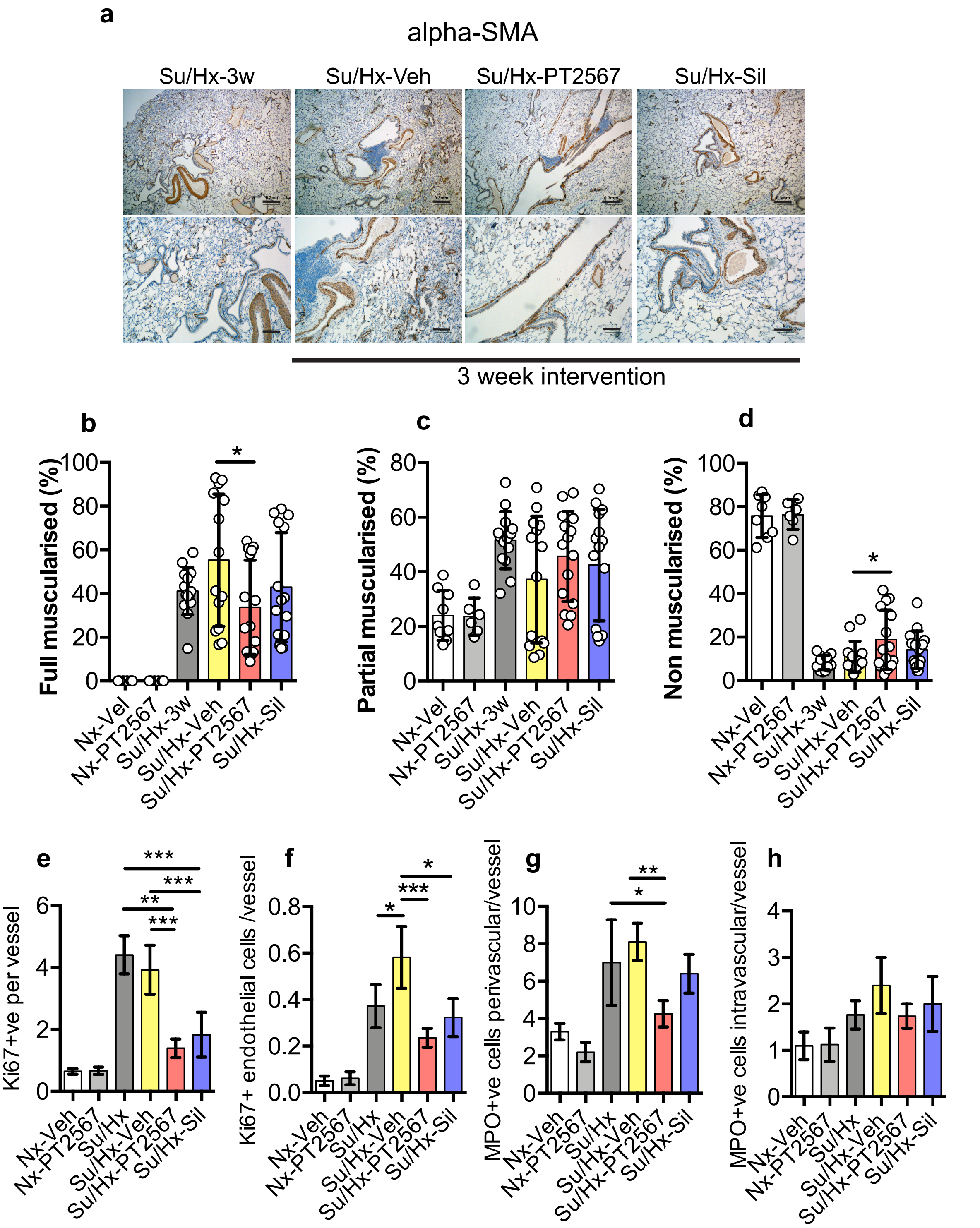

Figure S6 PT2567 intervention reduces pulmonary vascular remodeling
a Representative photomicrographs showing histological analysis of αSMA in lung sections. Scale bar on 0.3mm.
b-d Quantification of lung histological sections stained with αSMA. Distal muscularisation process characterised as (b) fully muscularised, (c) partially muscularised or (d) non-muscularised. Data information mean ± SD Nx/Veh n=8, SuHx-3w n=12, SuHx-Veh n=15, SuHx-PT2567 n=11, SuHx-sildenafil n=14.
e-f Bar chart of the number of (e) Ki67+ nuclei per vessel, (f) Ki67+ endothelial cells per vessel
g-h Bar chart of MPO immuno-positive cells perivascular (**g**) and intravascular (**h**) Data information mean ± SD Nx/Veh n=3, Nx/PT2567 n=3, SuHx-3w n=3, SuHx-Veh n=4, SuHx-PT2567 n=5, SuHx-sildenafil n=4. *p<0.05 **p<0.01 ***p<0.001 (one-way ANOVA)

Lung gene expression

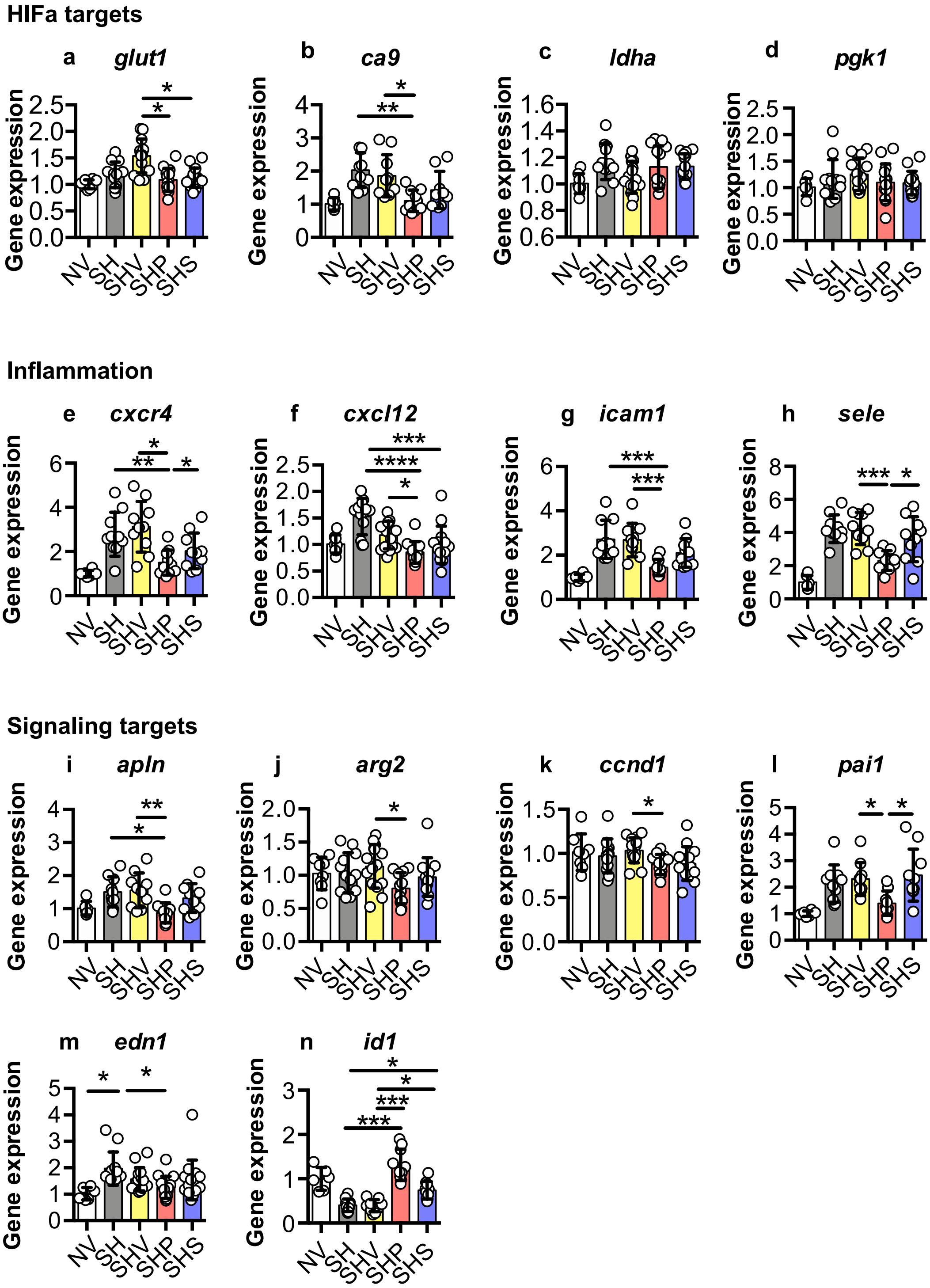

Figure S7 Inhibition of HIF2a with PT2567 modulates the expression of lung gene from Su/Hx rats
a-n qPCR analysis of whole lung samples (a) glut1, (b) ca9 (c) ldha, (d) pgk1, (e) cxcr4, (f) cxcl12, (g) icam1, (h) sele, (i) apln, (j) arg2, (k) ccnd1, (l) pai1, (m) edn1, (n) id1.
Data information mean ± SD rat Su5416/Hx intervention model (Nx/Veh n=8, Su/Hx-3w n=12, Su/Hx-Veh n=15, Su/Hx-PT2567 n=11, Su/Hx-sildenafil n=14). Data show fold change in gene expression relative to Nx/Veh control rats.*p<0.05, **p<0.001, ***p<0.001, ****p<0.0001(one-way ANOVA)
NV normoxia-vehicle, SH Sugen hypoxia 3 weeks, SHV Sugen hypoxia vehicle, SHP Sugen hypoxia PT2567 SHS Sugen hypoxia sildenafil

Right Ventricle gene expression

Structural Targets

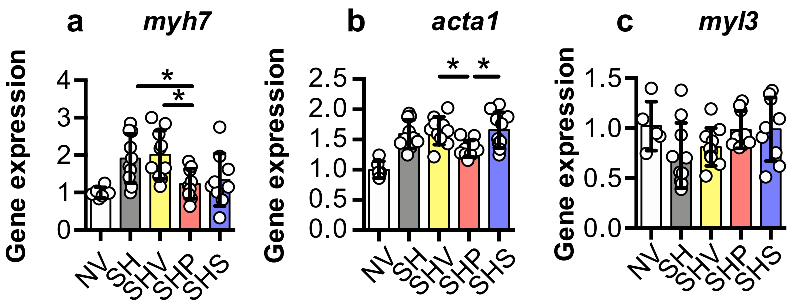

Inflammation targets

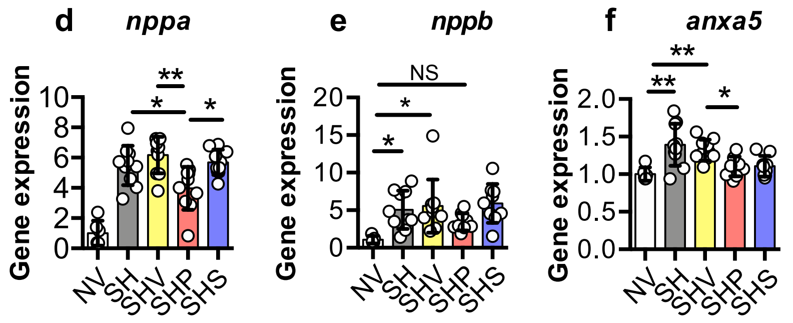

Fibrosis targets

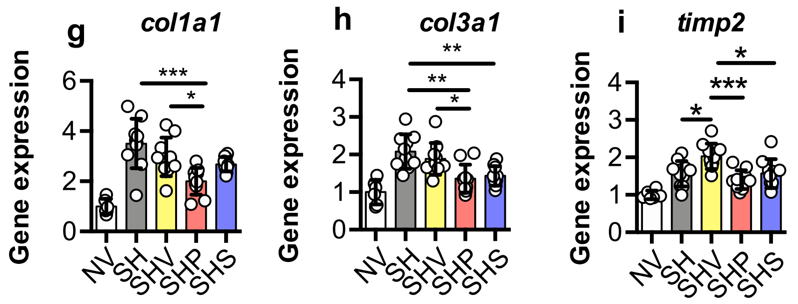

Figure S8 PT2567 modulates cardiac target gene expression in Su/Hx rats
a-i qPCR analysis of RV samples (a) myh7, (b) myl3, (c) acta1, (d) nppa, (e) nppb, (f) anxa5, (g) col1a1, (h) col3a1, (i) timp2.
Data information mean ± SD rat Su/Hx intervention model (Nx/Veh n=8, Su/Hx-3w n=12, Su/Hx-Veh n=15, Su/Hx-PT2567 n=11, Su/Hx-sildenafil n=14). Data show fold change in gene expression relative to Nx/Veh control rats.*p<0.05, **p<0.001, ***p<0.0001 (one-way ANOVA)
NV normoxia-vehicle, SH Sugen hypoxia 3 weeks, SHV Sugen hypoxia vehicle, SHP Sugen hypoxia PT2567
SHS Sugen hypoxia sildenafil

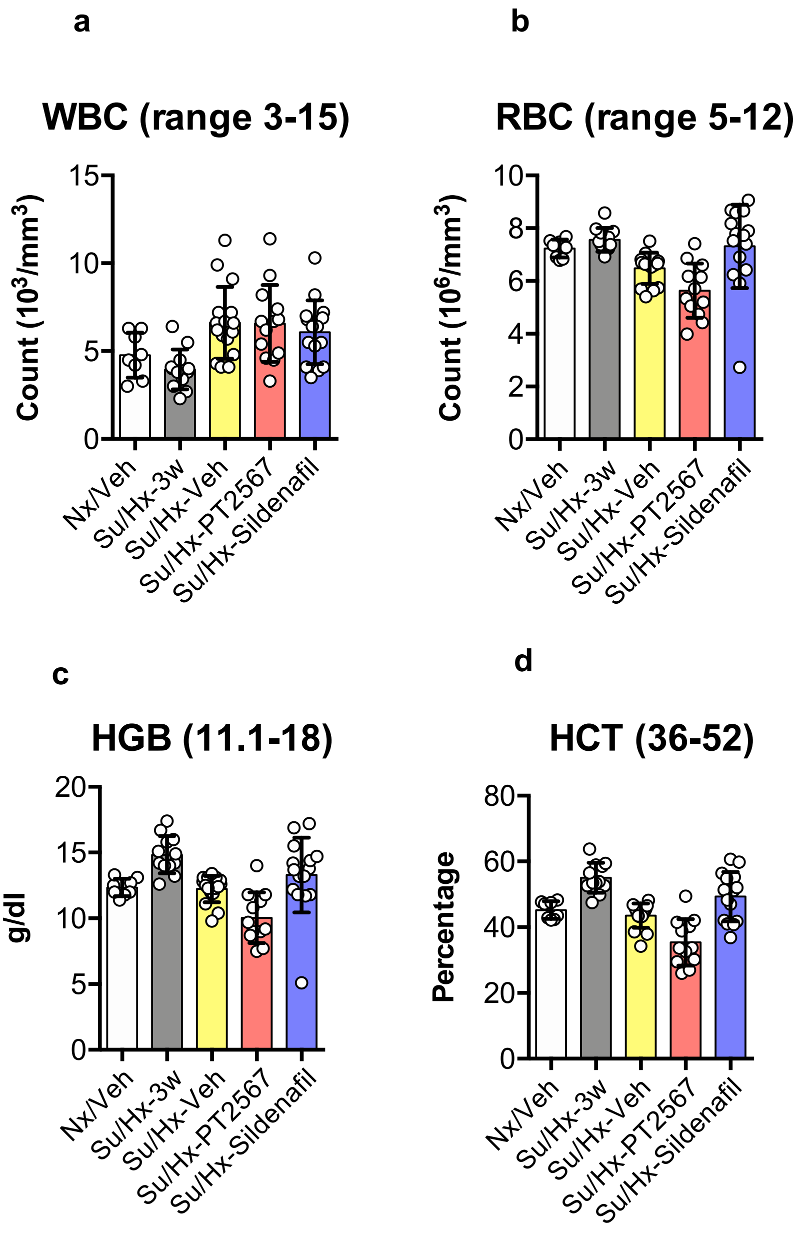

Figure S9. HIF2α inhibitor decreases RBC, HGB and HCT to lower physiological counts.
a-d Whole blood analysis (a) WBC, (b) RBC, (c) HGB and (d) HCT
Data information mean ± SD (Nx/Veh n=8, Su/Hx-3w n=12, Su/Hx-Veh n=15, Su/Hx-PT2567 n=11, Su/Hx-sildenafil n=14).

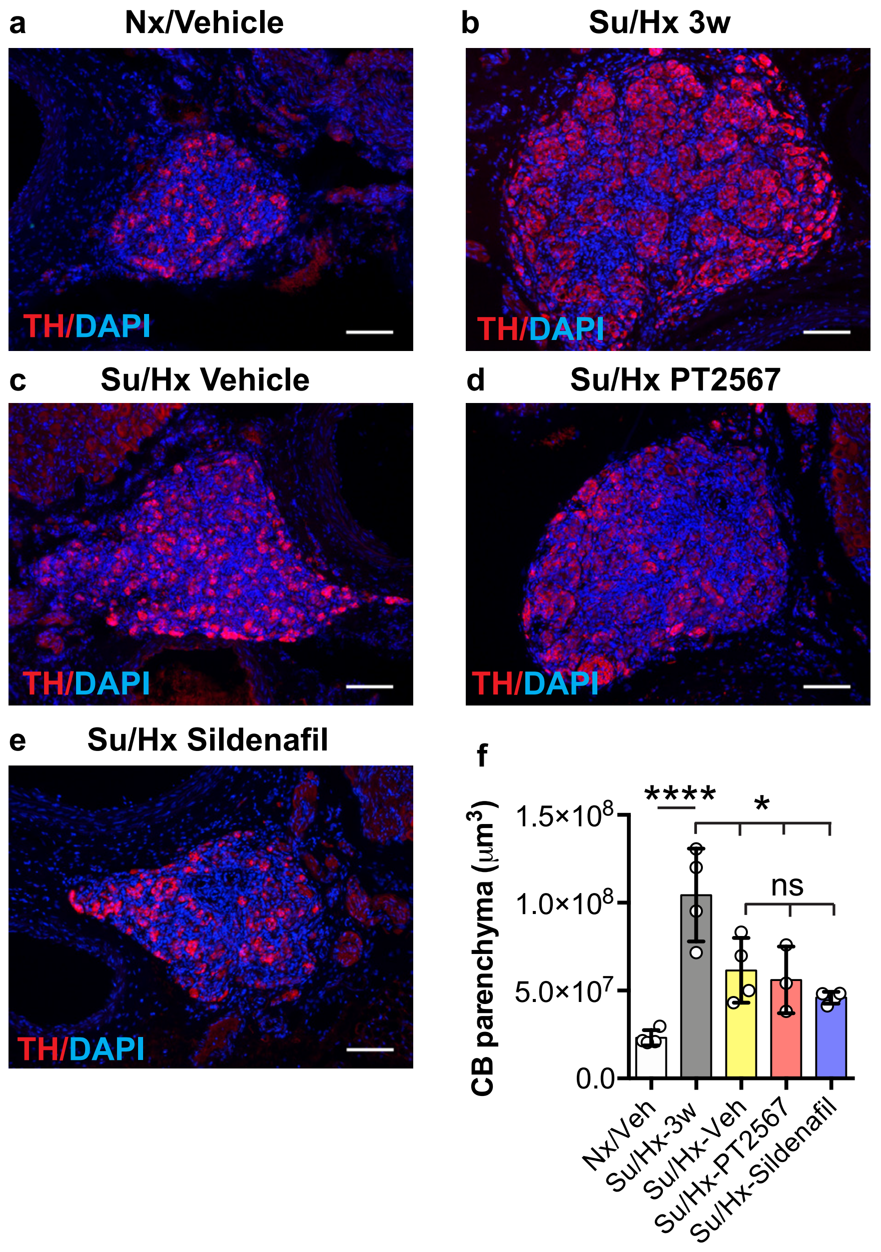

Figure S10. PT2567 does not alter carotid body morphology.
a-e Tyrosine hydroxylase (TH) immunostaining of the carotid bifurcation from rats exposed to the indicated treatments. Scale bars: 100 μm.
f CB volume quantification across the treatments.
Data information mean ±SEM Nx/Veh n=4, SuHx 3W n=4, SuHx Veh n=4, SuHx PT2567 n=3, SuHx sildenafil n=4. ****$p<0.0001$, *$p<0.05$, ns = non-significant (T-Test).

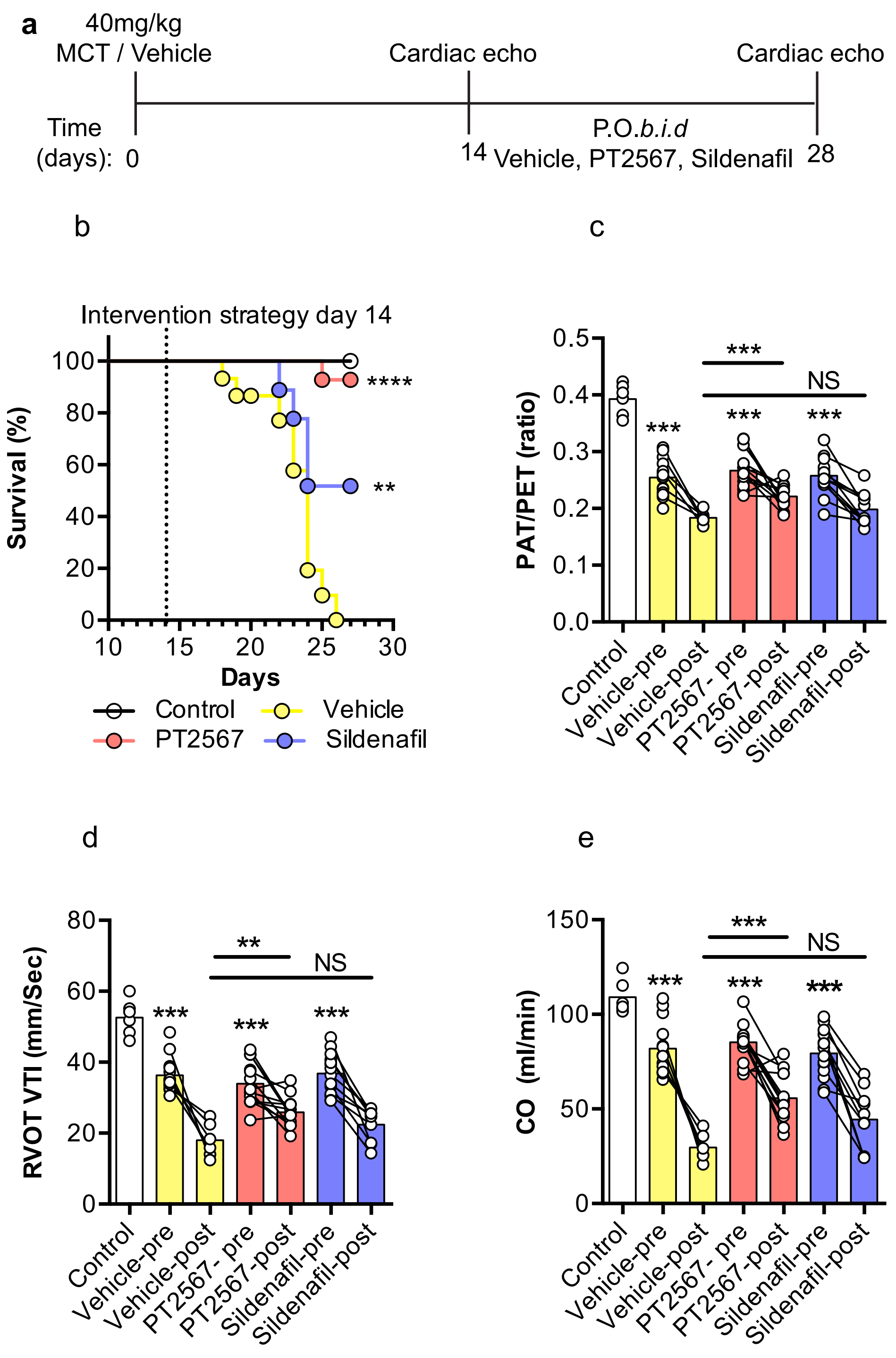

Figure S11. PT2567 treatment promotes survival in monocrotaline PH model.
(a) Line diagram showing the experimental time line of monocrotaline (MCT) rat PH model,
(b) PT2567 intervention inceased the survival rate of MCT treated animals when compared to sildenafil and vehicle controls. (c-e) Cardiac echo analyses pre- and post-intervention identified that PT2567 treatment reduced cardiac dysfunction, (c) PAT/PET ratio, (d) RVOT-VTI (e) cardiac output (CO) Kaplan-meier survival analysis assessed by log-rank (Mantel-Cox) test Data information, mean **p<0.001, ***p<0.0001 ****p<0.00001(one-way ANOVA)

Macias et al

Figure S12

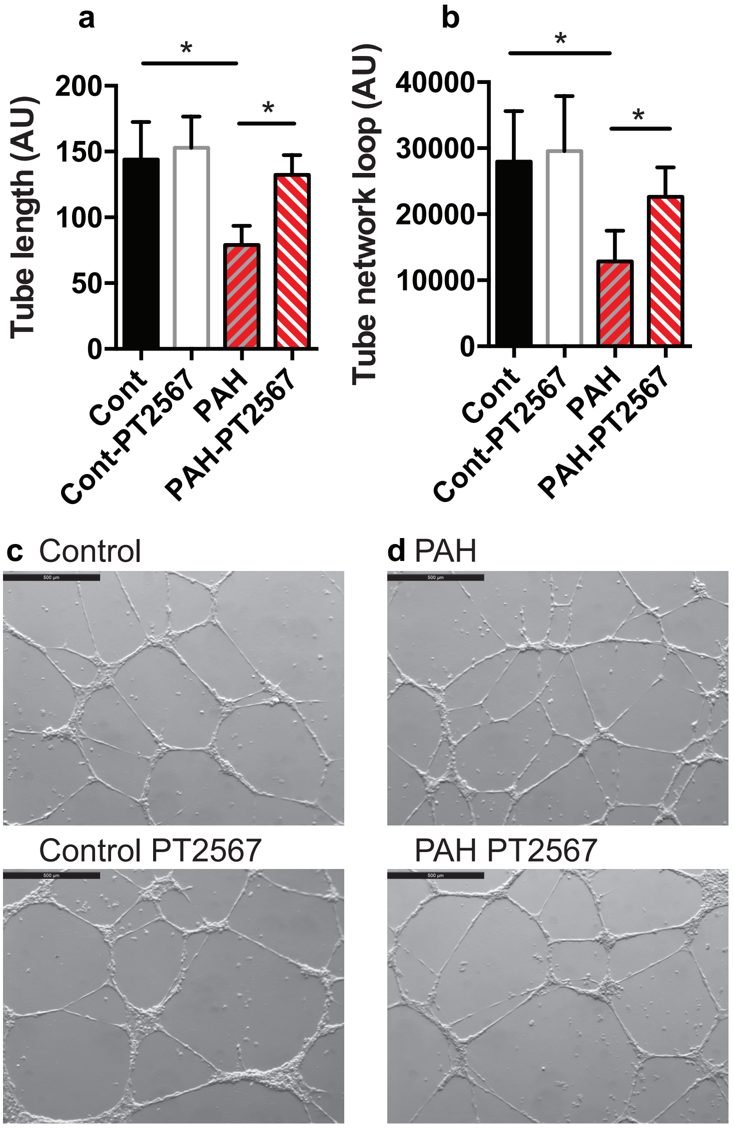

Figure S12. **PT2567 modulates aberant BOEC tube/network formation**
a Analysis of BOEC tube lengh between branch point. and b Measurment of BOEC network loop size following treatment with vehicle or PT2567 (1uM). Analysis was completed 20hr after plating BOECs.
c-d Representative photomicrographs of BOEC network formation assay from c, healthy control and d, PAH patient treated with vehicle of PT2567 (1uM)
