# Supplementary material for: Targeting HIF2α-ARNT hetero-dimerisation as a novel therapeutic strategy for pulmonary arterial hypertension
Source: Eur Respir J. 2021 Mar 4;57(3):1902061. doi: 10.1183/13993003.02061-2019 (PMC7930471; doi:10.1183/13993003.02061-2019)
Supplement: Supplementary file 2 [file ERJ-02061-2019_Supplementary_tables.pdf]

Supplemental tables 1a Prevention-protocol pulmonary histology assessment

Group	Animal	Perivascular/vascular inflammation	Perivascular fibrosis	Smooth muscle hypertrophy
1 Vehicle	1	0	0	2
	2	2	1	4
	3	1	1	4
	4	0	0	4
	5	1	1	4
	6	2	1	4
	7	1	1	4
	**Mean score**	**5.42**		

Group	Animal	Perivascular/vascular inflammation	Perivascular fibrosis	Smooth muscle hypertrophy
2 PT2567 100mg /kg	1	0	0	2
	2	1	0	3
	3	1	0	3
	4	0	0	2
	5	0	0	3
	6	1	1	4
	7	0	0	3
	8	1	1	4
	9	0	0	3
	**Mean Score**	**3.66**		

Group	Animal	Perivascular/vascular inflammation	Perivascular fibrosis	Smooth muscle hypertrophy
3 PT2567 300mg /kg	1	0	0	2
	2	0	1	4
	3	1	1	3
	4	0	0	2
	5	0	0	2
	6	0	0	2
	7	0	0	2
	8	0	0	2
	9	0	0	1
	**Mean Score**	**2.55**		

Group	Animal	Perivascular/vascular inflammation	Perivascular fibrosis	Smooth muscle hypertrophy
4 Sildena fil 30mg /kg	1	0	0	2
	2	0	0	3
	3	1	0	4
	4	0	0	3
	5	0	0	4
	6	1	1	4
	7	2	0	4
	8	0	1	3
	9	1	0	4
	10	0	0	3
	**Mean Score**	**4.00**		
5 Nx Control	1	0	0	0
	2	0	0	0
	**Mean Score**	**0**		

The number represents the grade (0=normal, 1=minimal, 2=mild, 3=moderate, 4=marked) for each lesion/finding

Supplemental tables 1b Intervention-protocol pulmonary histology assessment

Group	Animal	Perivascular/vascular inflammation	Perivascular fibrosis	Smooth muscle hypertrophy
1 Nx-Vehicle	1	0	0	0
	2	0	0	0
	3	0	0	1
	4	1	0	1
	5	0	1	0
	6	0	0	0
	**Mean score**	**0.66**		

Group	Animal	Perivascular/vascular inflammation	Perivascular fibrosis	Smooth muscle hypertrophy
2 Su/Hx 3W	1	3	2	2
	2	2	1	2
	3	2	2	3
	4	2	1	2
	5	2	1	2
	6	3	2	3
	7	2	2	2
	8	2	2	2
	9	3	3	3
	10	3	2	3
	**Mean Score**	**5.7**		

Group	Animal	Perivascular/vascular inflammation	Perivascular fibrosis	Smooth muscle hypertrophy
3 Su/Hx Vehicle	1	3	3	4
	2	3	3	2
	3	4	3	4
	4	2	2	3
	5	4	3	2
	6	2	2	2
	7	4	3	4
	8	2	3	4
	9	2	2	3
	10	3	3	4
	**Mean Score**	**8.9**		

Group	Animal	Perivascular/vascular inflammation	Perivascular fibrosis	Smooth muscle hypertrophy
4 Su/Hx PT2567 100mg/kg	1	2	1	1
	2	1	1	1
	3	1	1	1
	4	2	2	1
	5	1	1	2
	6	0	1	1
	7	1	1	1
	8	2	1	2
	9	3	2	3
	10	3	3	4
	**Mean Score**	**4.7**		

Group	Animal	Perivascular/vascular inflammation	Perivascular fibrosis	Smooth muscle hypertrophy
5 Su/H Sildenafil 30mg/kg	1	2	2	2
	2	2	1	1
	3	4	2	3
	4	3	2	2
	5	2	2	3
	6	2	2	2
	7	2	2	2

	8	2	1	1
	9	1	1	2
	10	3	2	2
	**Mean Score**	**5.9**		

The number represents the grade (0=normal, 1=minimal, 2=mild, 3=moderate, 4=marked) for each lesion/finding
