# Supplementary material for: Targeting HIF2α-ARNT hetero-dimerisation as a novel therapeutic strategy for pulmonary arterial hypertension
Source: Eur Respir J. 2021 Mar 4;57(3):1902061. doi: 10.1183/13993003.02061-2019 (PMC7930471; doi:10.1183/13993003.02061-2019)
Supplement: Supplementary file 3 [file ERJ-02061-2019.Shareable.pdf]

SHAREABLE PDF

Targeting HIF2α-ARNT hetero-dimerisation as a novel therapeutic strategy for pulmonary arterial hypertension

David Macias [1,6], Stephen Moore[2,6], Alexi Crosby[2], Mark Southwood[3], Xinlin Du[4], Huiling Tan[4], Shanhai Xie[4], Arlette Vassallo [2], Alexander J.T. Wood [2], Eli M. Wallace[4] and Andrew S. Cowburn [2,5]

Affiliations: [1]CRUK Cambridge Centre Early Detection Programme, Dept of Oncology, Hutchison/MRC Research Centre, University of Cambridge, Cambridge, UK. [2]Dept of Medicine, University of Cambridge, Cambridge, UK. [3]Dept of Pathology, Papworth Hospital National Health Service Foundation Trust, Cambridge, UK. [4]Peloton Therapeutics Inc. (a subsidiary of Merck & Co., Inc., Kenilworth, NJ, USA), Dallas, TX, USA. [5]National Heart and Lung Institute, Imperial College London, London, UK. [6]Both authors contributed equally.

Correspondence: Andrew S. Cowburn, National Heart and Lung Institute, Imperial College London, ICTEM Building, Hammersmith Campus, Du Cane Road, London, W12 0NN, UK. E-mail: acowburn@ic.ac.uk

@ERSpublications
PAH is a debilitating disease with no cure. There is an unmet need for new transformative therapies. Targeting HIF2α function through inhibiting ARNT hetero-dimerisation reduces many clinical symptoms associated with established PH disease in animals. https://bit.ly/3jHK8PS

Cite this article as: Macias D, Moore S, Crosby A, *et al.* Targeting HIF2α-ARNT hetero-dimerisation as a novel therapeutic strategy for pulmonary arterial hypertension. *Eur Respir J* 2021; 57: 1902061 [https://doi.org/10.1183/13993003.02061-2019].

This single-page version can be shared freely online.

ABSTRACT Pulmonary arterial hypertension (PAH) is a destructive disease of the pulmonary vasculature often leading to right heart failure and death. Current therapeutic intervention strategies only slow disease progression. The role of aberrant hypoxia-inducible factor (HIF)2α stability and function in the initiation and development of pulmonary hypertension (PH) has been an area of intense interest for nearly two decades.

Here we determine the effect of a novel HIF2α inhibitor (PT2567) on PH disease initiation and progression, using two pre-clinical models of PH. Haemodynamic measurements were performed, followed by collection of heart, lung and blood for pathological, gene expression and biochemical analysis. Blood outgrowth endothelial cells from idiopathic PAH patients were used to determine the impact of HIF2α-inhibition on endothelial function.

Global inhibition of HIF2a reduced pulmonary vascular haemodynamics and pulmonary vascular remodelling in both su5416/hypoxia prevention and intervention models. PT2567 intervention reduced the expression of PH-associated target genes in both lung and cardiac tissues and restored plasma nitrite concentration. Treatment of monocrotaline-exposed rodents with PT2567 reduced the impact on cardiovascular haemodynamics and promoted a survival advantage. *In vitro*, loss of HIF2α signalling in human pulmonary arterial endothelial cells suppresses target genes associated with inflammation, and PT2567 reduced the hyperproliferative phenotype and overactive arginase activity in blood outgrowth endothelial cells from idiopathic PAH patients. These data suggest that targeting HIF2α hetero-dimerisation with an orally bioavailable compound could offer a new therapeutic approach for PAH. Future studies are required to determine the role of HIF in the heterogeneous PAH population.

Copyright ©ERS 2021. This version is distributed under the terms of the Creative Commons Attribution Licence 4.0.
